# Supplementary material for: Full-color, time-valve controllable and Janus-type long-persistent luminescence from all-inorganic halide perovskites
Source: Nat Commun. 2024 Jun 20;15:5281. doi: 10.1038/s41467-024-49654-7 (PMC11190143; doi:10.1038/s41467-024-49654-7)
Supplement: Supplementary file 1 — Supplementary Information [file 41467_2024_49654_MOESM1_ESM.pdf]

Supplementary Information

Full-color, time-valve controllable and Janus-type long-persistent luminescence from all-inorganic halide perovskites

Chen et al.

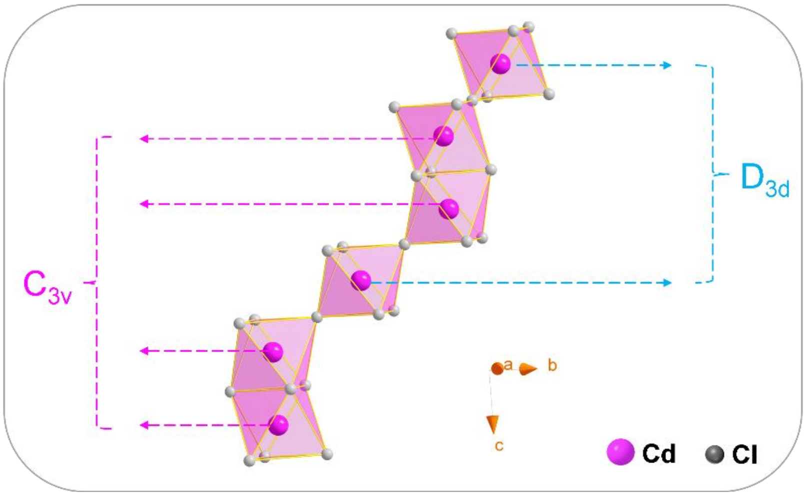

Supplementary Fig. 1. Crystal symmetry of $CsCdCl_3$. The crystal structure with the symmetry of $CsCdCl_3$ perovskites by removing the Cs atoms.

Supplementary Table 1 Crystallographic data for pure $CsCdCl_3$ perovskites at 100 K.

	**$CsCdCl_3$**
Formula	$CsCdCl_3$
Crystal system	hexagonal
Space group	$P\ 6_3/mmc$
a/Å	7.3797(4)
b/Å	7.3797(4)
c/Å	18.3778(10)
α/°	90
β/°	90
γ/°	120
Volume/$Å^3$	866.76(11)
Z	7.25
DC (g/cm^3)	4.042
μ/mm^{-1}	11.210
F(000)	924.0
Index ranges	$-8 \leq h \leq 8$, $-8 \leq k \leq 7$, $-21 \leq l \leq 21$

Reflections collected	5535
Unique reflections	329
R_{int}	0.0600
GOF on F^2	1.214
R_1, wR [I>=2σ (I)]	0.0182, 0.0420
R_1, wR [all data]	0.0217, 0.0439

[a] $R_1 = \Sigma \| F_o| - |F_c \| / |F_o|$. [b] $wR_2 = \{\Sigma[w(F_o^2 - F_c^2)^2] / \Sigma w(F_o^2)^2\}^{1/2}$.

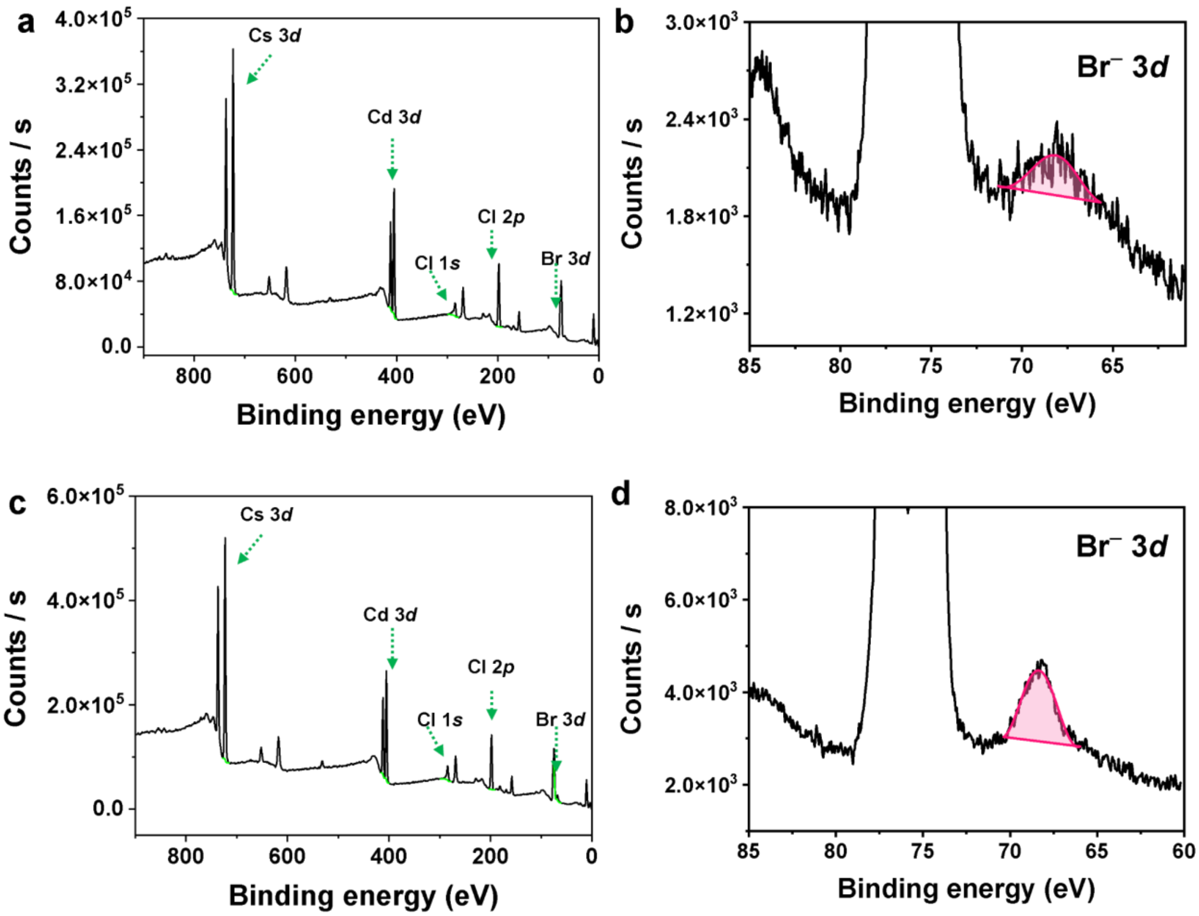

Supplementary Fig. 2. XPS analyses. Full XPS spectra **a** $CsCdCl_3$:0.8%Br and **c** $CsCdCl_3$:10%Br. High-resolution XPS spectra of Br 3*d* level in **b** $CsCdCl_3$:0.8%Br and **d** $CsCdCl_3$:10%Br.

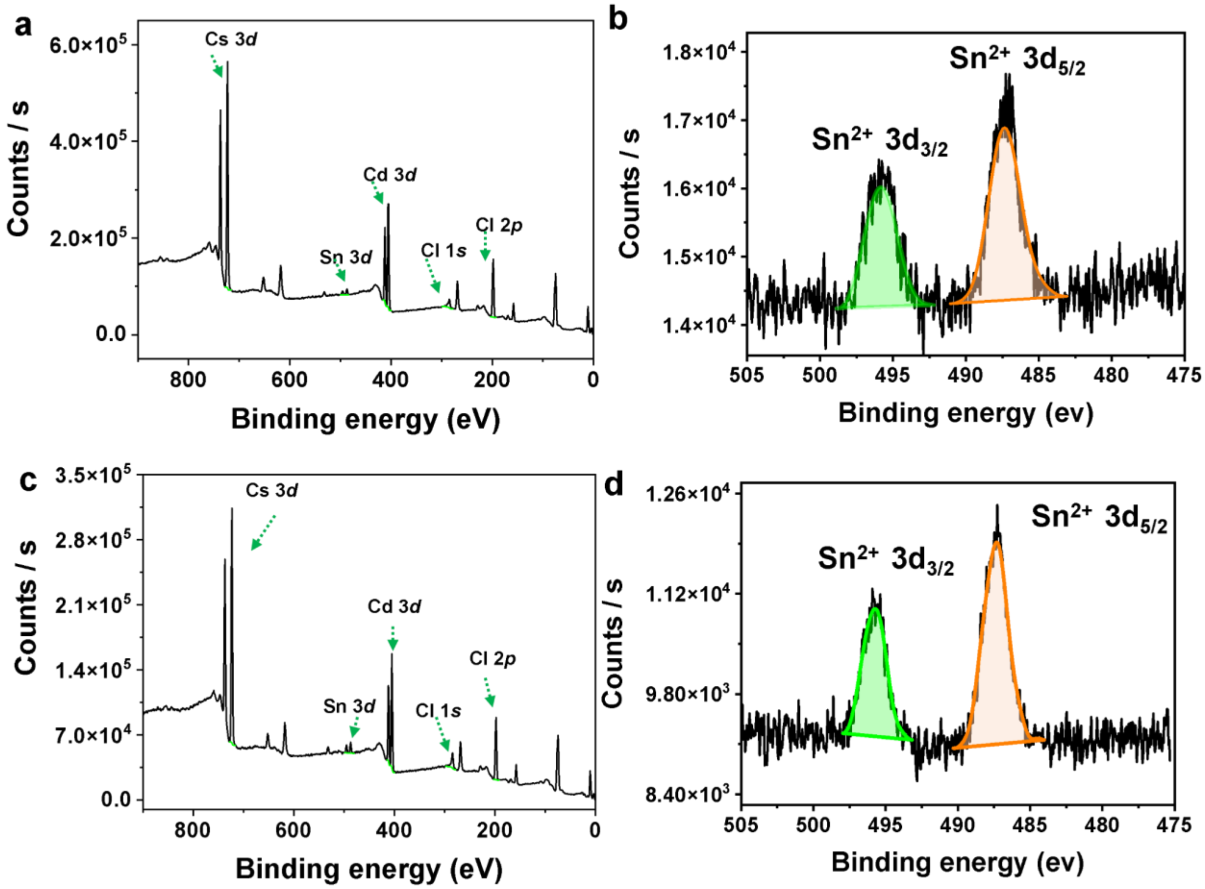

Supplementary Fig 3. XPS analyses. Full XPS spectra **a** $CsCdCl_3$:3%Sn and **c** $CsCdCl_3$:10%Sn. High-resolution XPS spectra of Sn 3*d* level in **b** $CsCdCl_3$:3%Sn and **d** $CsCdCl_3$:10%Sn.

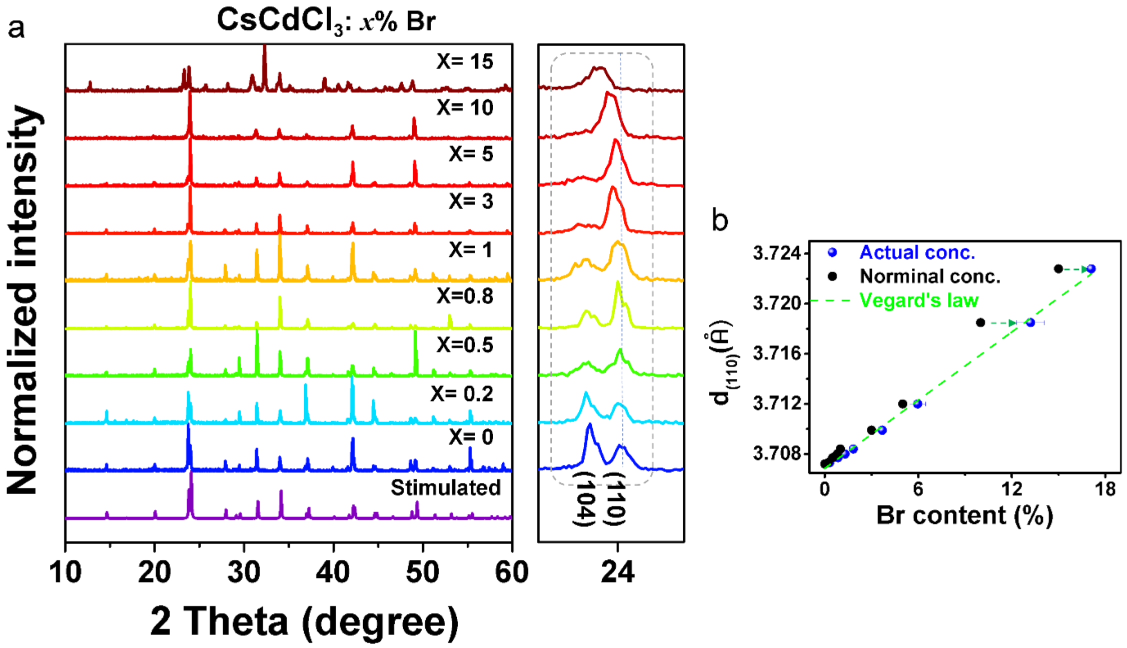

Supplementary Fig. 4. XRD analyses. a XRD patterns of the sample $CsCdCl_3$:*x*%Br (*x*=0, 0.2, 0.5, 0.8, 1, 3, 5, 10, 15) and the magnified peaks of (110) plane suggested a lattice expansion after Br^- doping. **b** Both nominal (black dot) and actual (blue dot) concentrations of Br^- ions were plotted against d-spacing of (110) plane as calculated based on XRD data. Note that the actual concentration was determined by averaging three measurements of energy dispersive spectroscopy (EDS), which echoed well with Vegard's law. Error bars on the x-axis indicate the standard deviation across N = 3 experimental measurements, with all points centered around the mean.

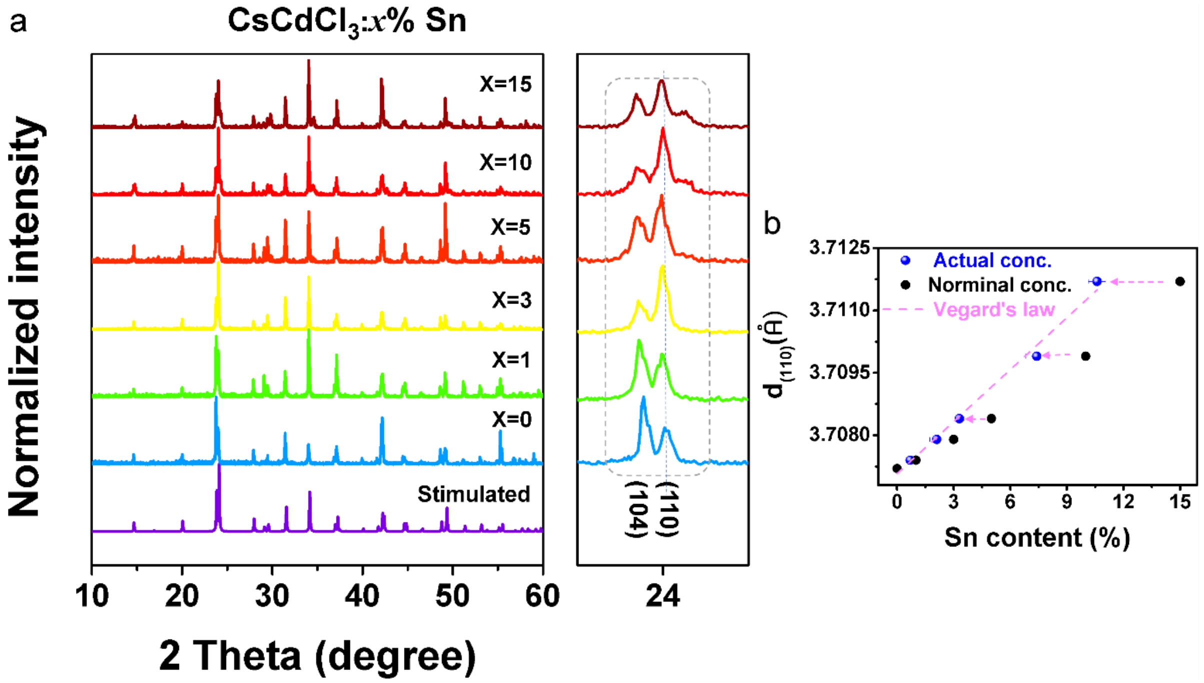

Supplementary Fig. 5. XRD analyses. a XRD patterns of the sample $CsCdCl_3$:*x*%Sn (*x*=0, 1, 3, 5, 10, 15) and the magnified peaks of (110) plane suggested a lattice expansion after Sn^{2+} doping. **b** Both nominal (black dot) and actual (blue dot) concentrations of Sn^{2+} ions were plotted against d-spacing of (110) plane as calculated based on XRD data. Note that the actual concentration was determined by averaging three measurements of energy dispersive spectroscopy (EDS), which echoed well with Vegard's law. Error bars on the x-axis indicate the standard deviation across N = 3 experimental measurements, with all points centered around the mean.

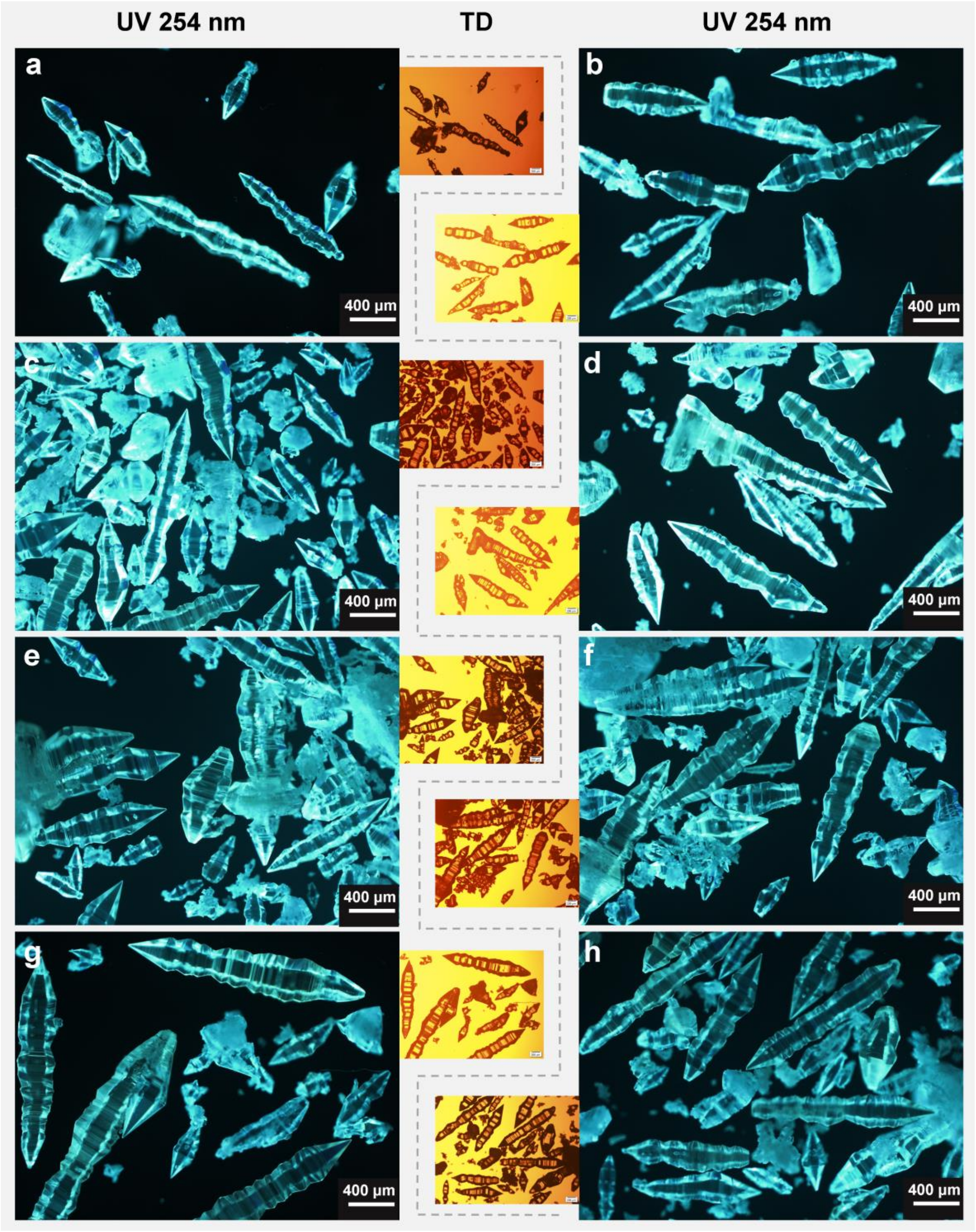

Supplementary Fig. 6. The fluorescence and bright field images of $CsCdCl_3$:*x*%Br, with **a** (0.2%Br), **b** (0.5%Br), **c** (0.8%Br), **d** (1%Br), **e** (3%Br), **f** (5%Br), **g** (10%Br) and **h** (15%Br).

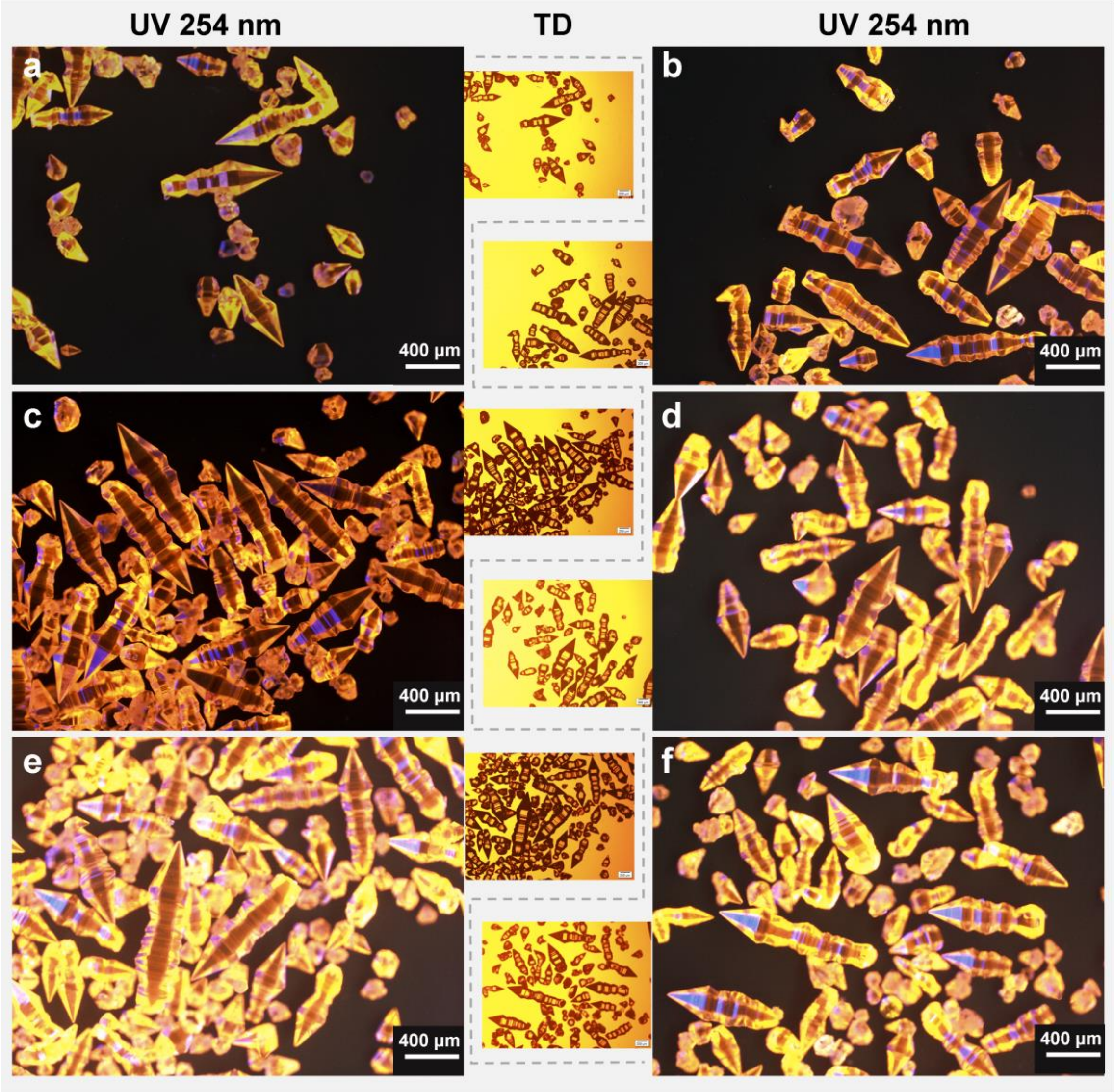

Supplementary Fig. 7. The fluorescence and bright field images of $CsCdCl_3$:*x*%Sn, **a** (0%Sn), **b** (1%Sn), **c** (3%Sn), **d** (5%Sn), **e** (10%Sn) and **f** (15%Sn).

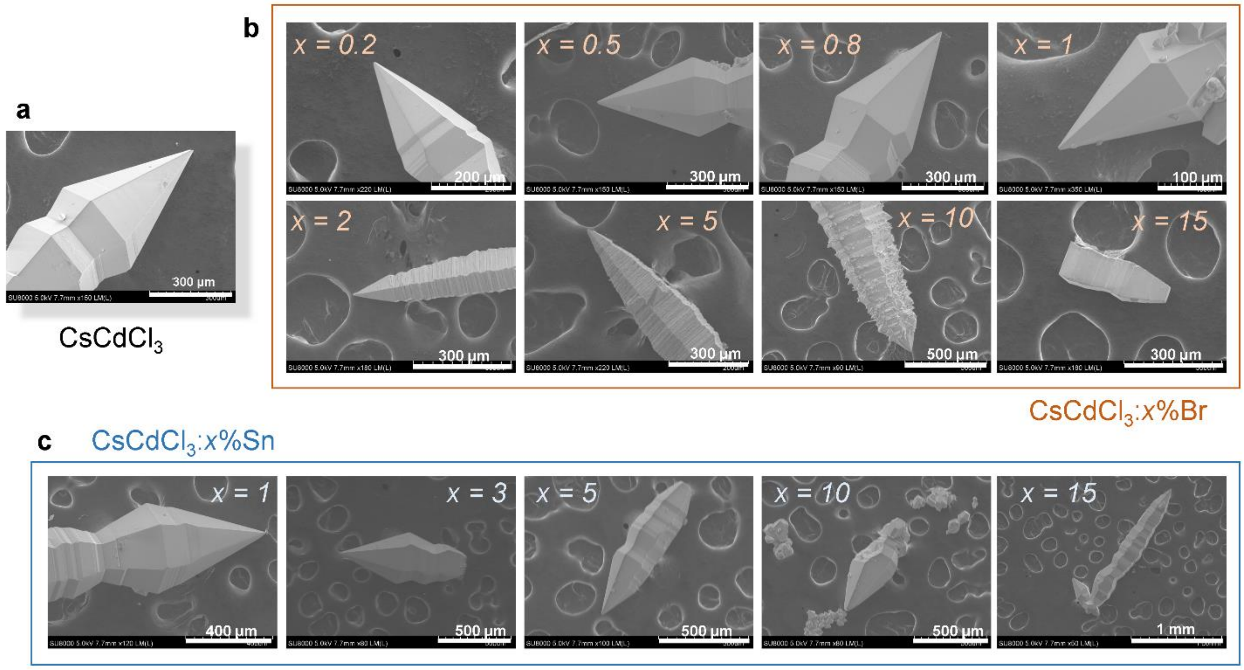

Supplementary Fig. 8. SEM images of single crystalline **a** $CsCdCl_3$, **b** $CsCdCl_3$:*x*%Br and **c** $CsCdCl_3$:*x*%Sn.

Supplementary Table 2 The actual doping concentration of Br^{-} ions corrected by Vegard's law (XRD) and EDS.

Norminal conc.	XRD conc. (110)	EDS	
0.2%	0.50%	0.18%/0.29%/0.46%	0.31%±0.12%
0.5%	0.94%	1.24%/0.75%/0.53%	0.84%±0.30%
0.8%	1.27%	1.31%/1.15%/1.44%	1.30%±0.12%
1.0%	1.71%	1.65%/1.82%/2.02%	1.83%±0.15%
3.0%	3.36%	3.95%/3.67%/3.45%	3.69%±0.20%
5.0%	5.67%	5.33%/5.98%/6.57%	5.96%±0.51%
10.0%	12.83%	12.08%/13.25%/14.27%	13.20%±0.89%
15.0%	17.56%	16.76%/17.40%/17.14%	17.10%±0.26%

Note: The left EDS column refers to the concentration determined through three measurements of energy dispersive spectroscopy (EDS), while the right column represents the averaged concentration and standard deviation derived from the left EDS column. Conc. refers to concentration.

Supplementary Table 3 The actual doping concentration of Sn^{2+} ions corrected by Vegard's law (XRD) and EDS.

Norminal conc.	XRD conc. (110)	EDS	
1.0%	0.77%	0.6%/0.5%/1.0%	0.7% ± 0.22%
3.0%	1.97%	2.4%/2.3%/1.6%	2.1% ± 0.36%
5.0%	3.17%	3.0%/3.6%/3.3%	3.3% ± 0.24%
10.0%	6.76%	6.1%/8.2%/7.9%	7.4% ± 0.59%
15.0%	11.15%	10.1%/9.6%/12.1%	10.6% ± 0.45%

Note: The left EDS column refers to the concentration determined through three measurements of energy dispersive spectroscopy (EDS), while the right column represents the averaged concentration and standard deviation derived from the left EDS column. Conc. refers to concentration.

Supplementary Table 4. The result of ICP-OES measurements for $CsCdCl_3$:10%Sn.

Cd/Sn molar feed ratio	Actual Cd concentration (μg/mL)	Actual Sn concentration (μg/mL)	Actual Cd-to-Sn molar rato
90.0% :10.0%	161.10	13.15	92.99% : 7.01%

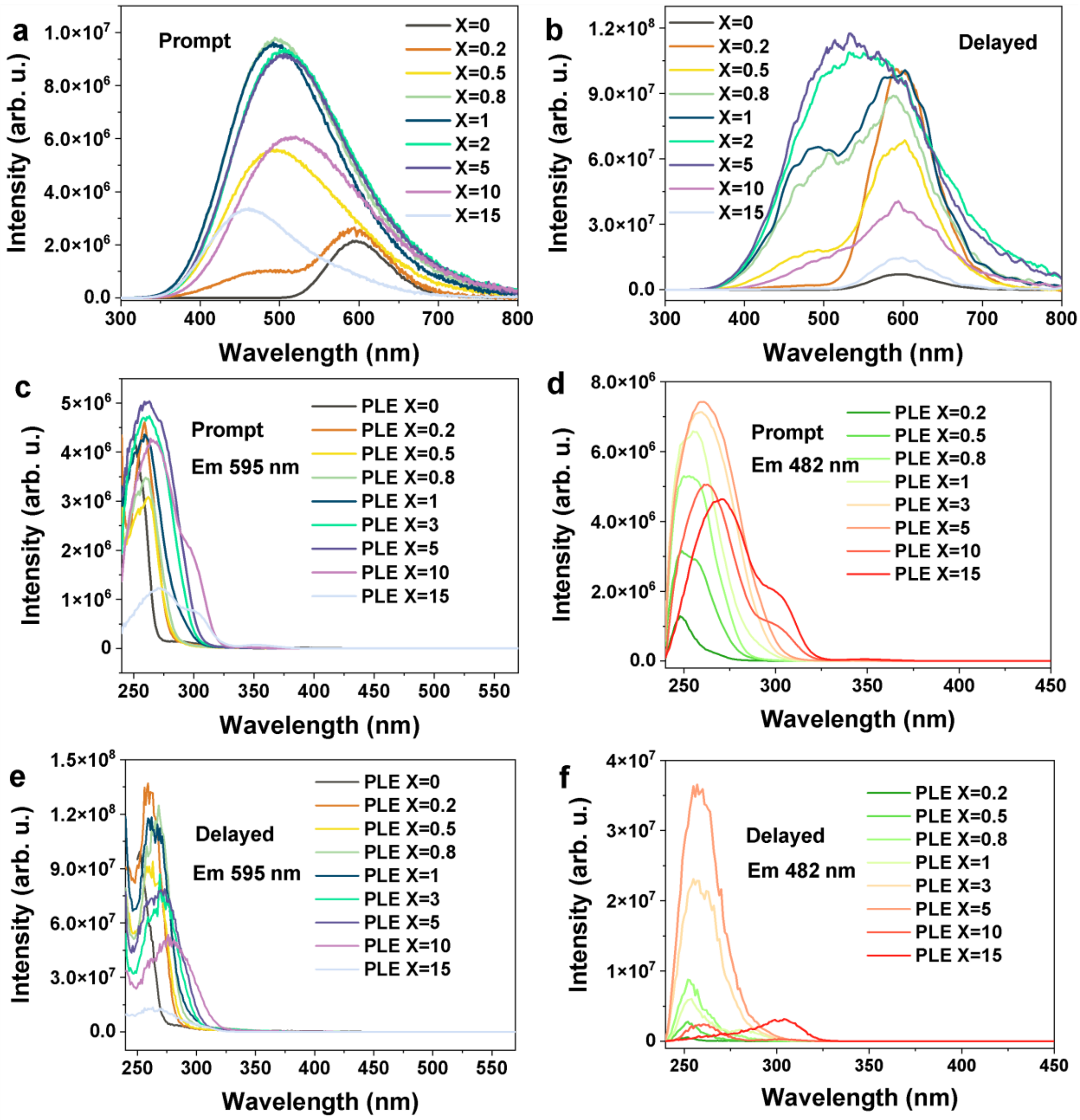

Supplementary Fig. 9. Photoluminescence spectral studies. a Prompt and **b** delayed emission (t_d = 1 ms) spectra of $CsCdCl_3$:*x*%Br under 254 nm excitation. The PLE spectra of $CsCdCl_3$:*x*%Br were monitored at **c** 595 nm and **d** 482 nm in prompt pattern. The PLE spectra of $CsCdCl_3$:*x*%Br were monitored at **e** 595 nm and **f** 482 nm in delayed pattern. x=0, 0.2, 0.5, 0.8, 1, 3, 5, 10 and 15.

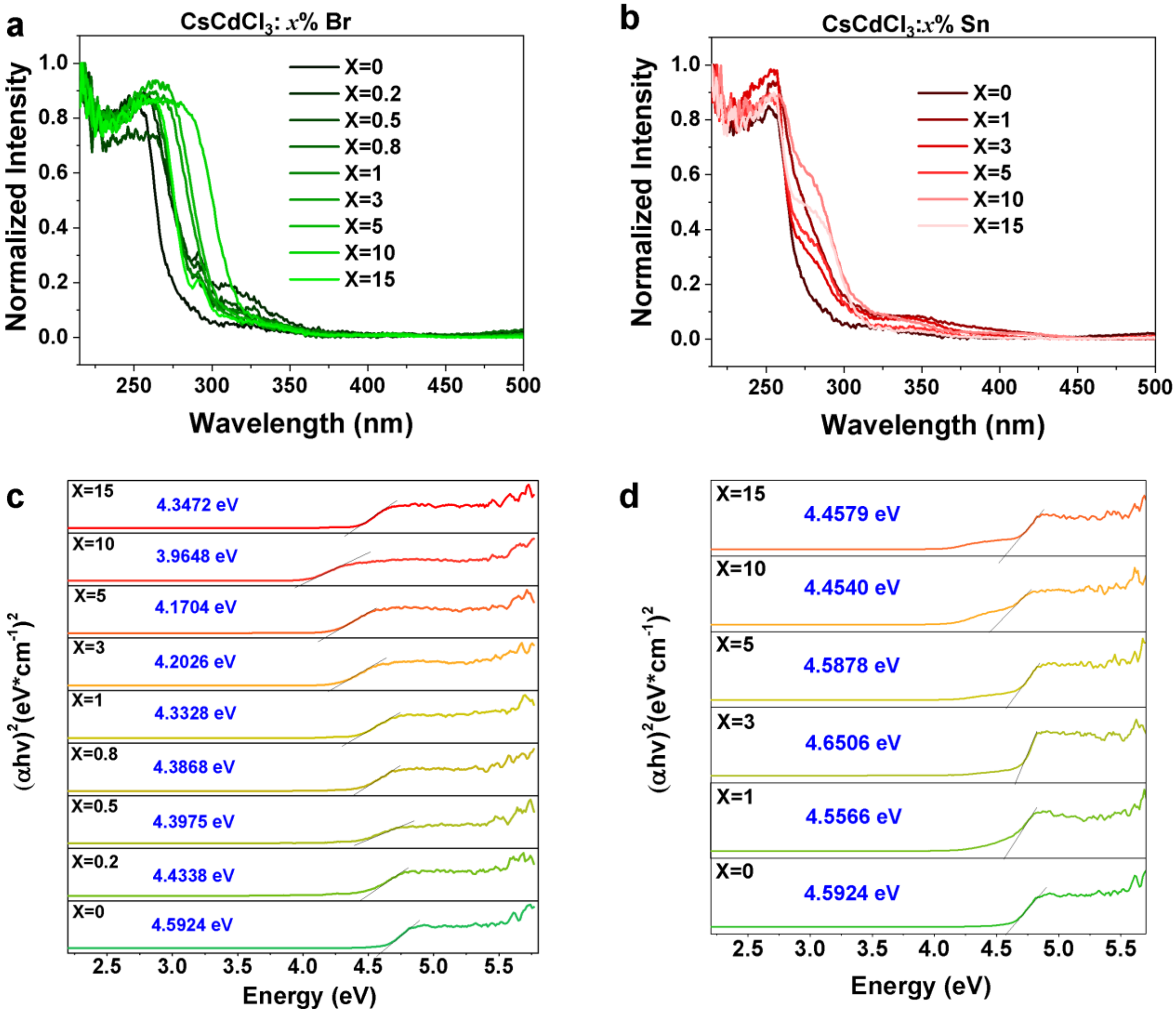

Supplementary Fig. 10 Solid-state UV-vis absorption spectra of **a** $CsCdCl_3$:*x*%Br (x=0, 0.2, 0.5, 0.8, 1, 3, 5, 10, 15) and **b** $CsCdCl_3$:*x*%Sn (x=0, 1, 3, 5, 10, 15). The corresponding Tauc plots of **c** $CsCdCl_3$:*x*%Br (x=0, 0.2, 0.5, 0.8, 1, 3, 5, 10, 15) and **d** $CsCdCl_3$:*x*%Sn (x=0, 1, 3, 5, 10, 15). The Tauc plot is a method to derive the optical gap. By measuring the absorption coefficient α(hν) and plotting $(\alpha h\nu)^2$ versus photon energy hν, the value for the optical gap (Tauc gap) is determined.

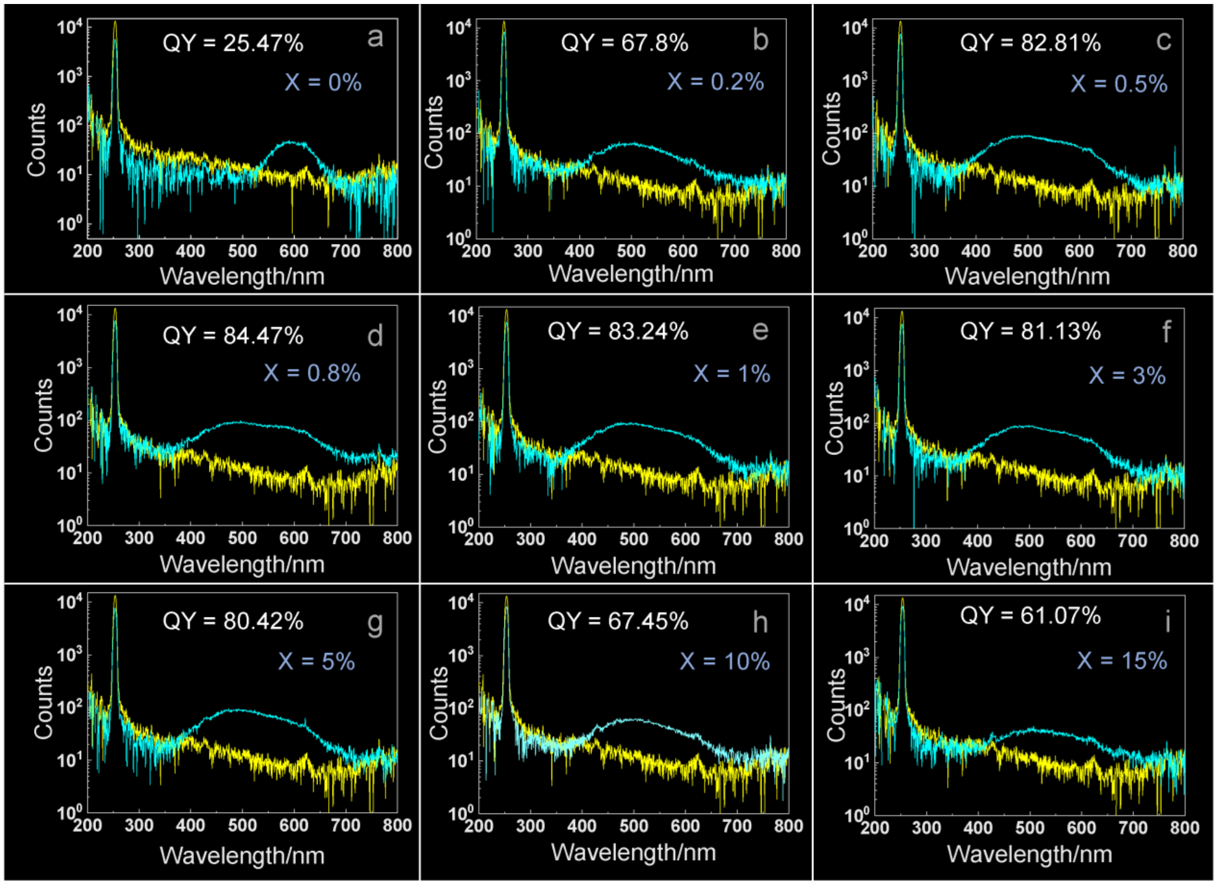

Supplementary Fig. 11 a-i Quantum yields of $CsCdCl_3$:*x*%Br (x=0, 0.2, 0.5, 0.8, 1, 3, 5, 10, 15).

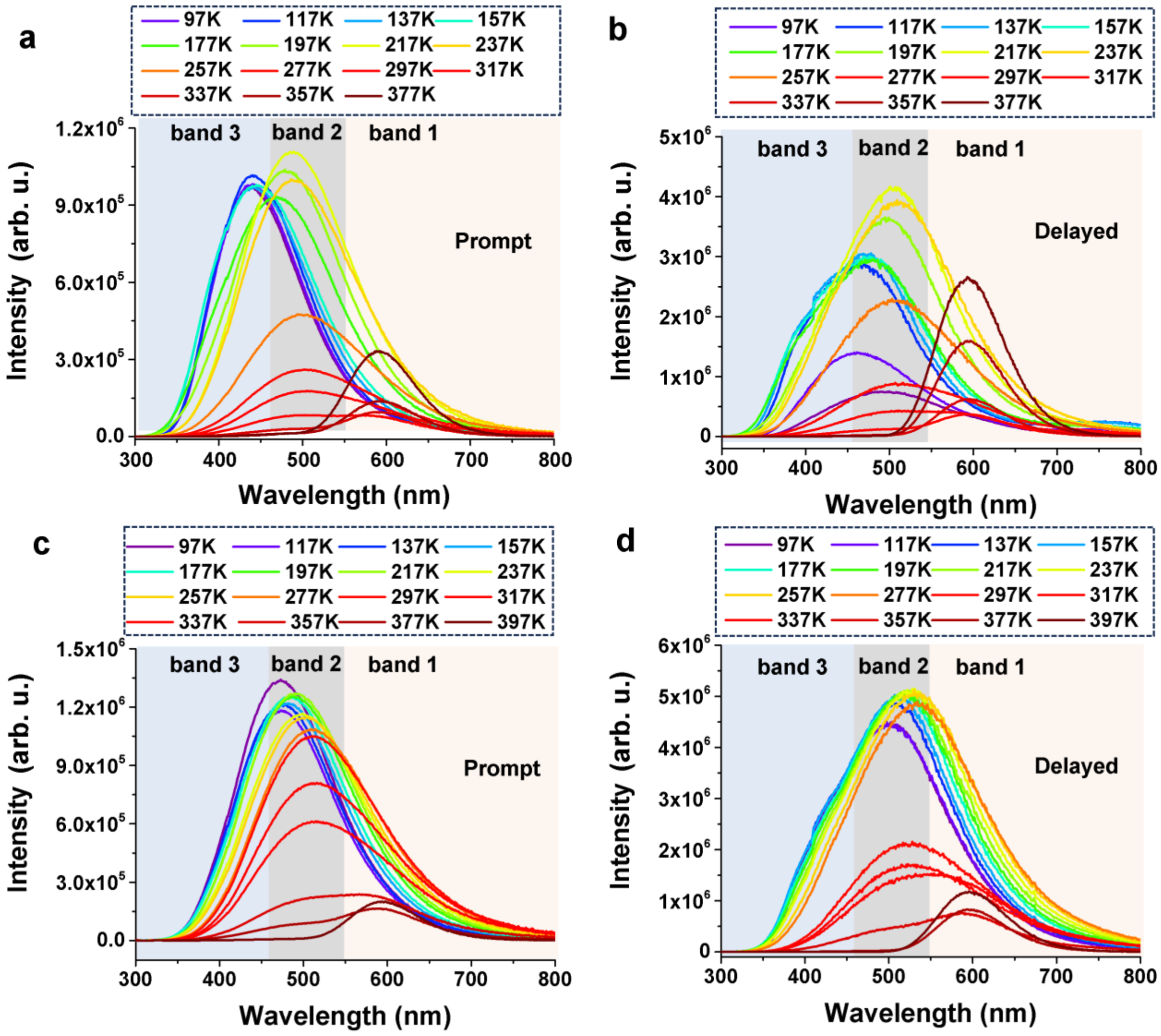

Supplementary Fig. 12. Temperature-dependent photoluminescence spectral studies. The PL spectra of $CsCdCl_3$:0.8%Br at different temperatures ranging from 97 to 377 K based on **a** prompt and **b** delayed (t_d = 1 ms) patterns under 254 nm excitation. The PL spectra of $CsCdCl_3$:10%Br at different temperatures ranging from 97 to 397 K based on **c** prompt and **d** delayed (t_d = 1 ms) patterns under 254 nm excitation.

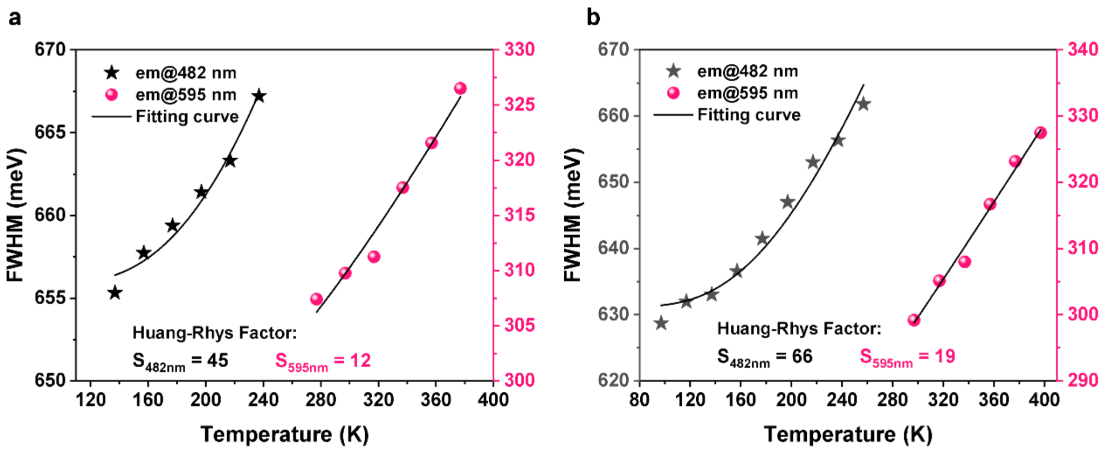

Supplementary Figs. 13. FWHM of **a** $CsCdCl_3$:0.8%Br and **b** $CsCdCl_3$:10%Br at 482 nm and 595 nm versus temperature form the delayed spectra of Supplementary Fig. 10, respectively.

The electron-phonon coupling effect of Br-doped $CsCdCl_3$ was discussed by fitting FWHM versus temperature according to follow Equation S1:

$$\mathrm{FWHM}=2.36\sqrt{S}\hbar\omega_{\mathrm{phonon}}\sqrt{\coth\frac{\hbar\omega_{\mathrm{phonon}}}{2k_BT}} \qquad \mathrm{S1}$$

Here, k_B is the Boltzmann constant, and S is the Huang-Rhys electron-phonon coupling parameter, and $\hbar\omega_{\mathrm{phonon}}$ is the effective phonon energy.

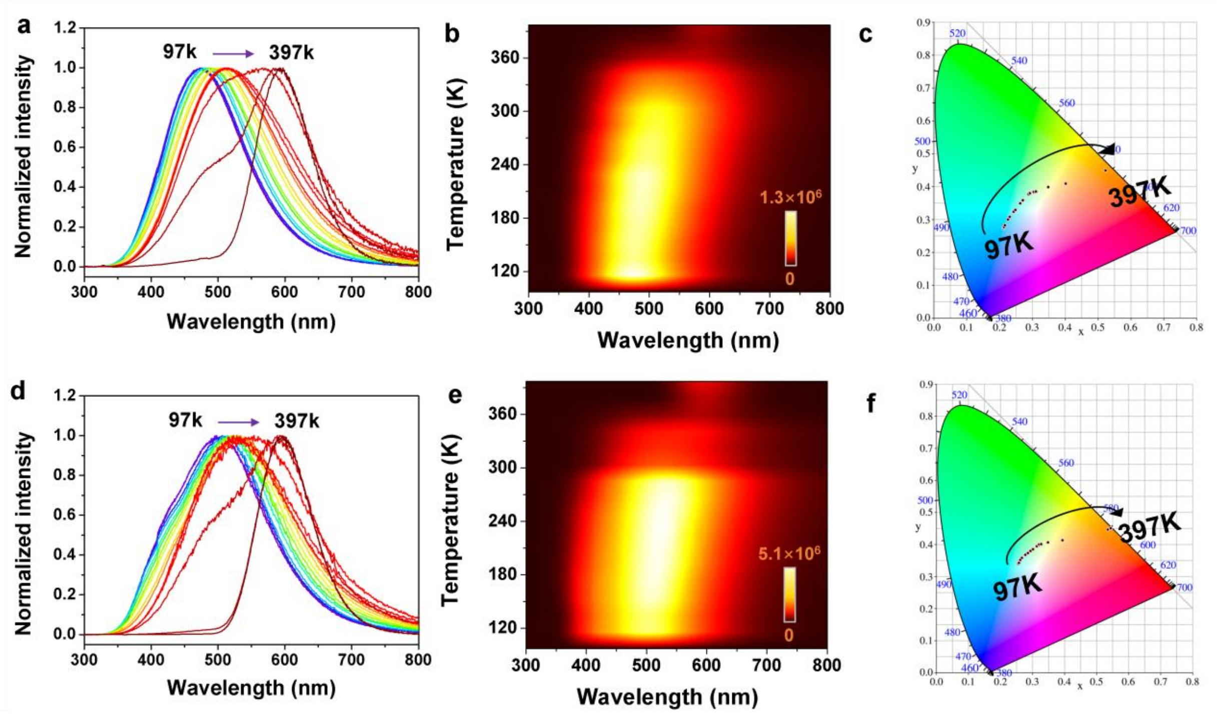

Supplementary Fig. 14. Temperature-dependent photoluminescence spectral studies. The normalized PL spectra of $CsCdCl_3$:10%Br at different temperatures ranging from 97 to 397 K based on **a** prompt and **d** delayed (t_d = 1 ms) patterns under 254 nm excitation. Pseudo color map of temperature dependent PL of $CsCdCl_3$:10%Br based on **b** prompt and **e** delayed (t_d = 1 ms) patterns under 254 nm excitation. CIE coordinate diagram of $CsCdCl_3$:10%Br in temperature - responsive **c** prompt and **f** delayed mode. Note: Due to limited space of the picture, the color gradient of curves in Supp. Fig 14 a,d only represents the change in emission peaks based on temperatures (the arrows from left to right: 97, 117, 137, 157, 177, 197, 217, 257, 277, 297, 317, 357, 377, and 397K, respectively), without any other special meaning.

Supplementary Table 5 Properties of LPL materials in recent years.

Compound	Categories and temperature of synthesis(°C)	excitation	LPL lifetime	Φ (%)	Time -dependent	Temperature -dependent	-excitation -dependent		anti-thermal quenching	Reference
							Forward	Reverse		
$CsCdCl_3$:0.8%Br	Perovskites,180	254 nm	>2000s	84.47	Yes	Yes	--	--	Yes	This work
$CsCdCl_3$:10%Sn	Perovskites,180	254 nm	>2000s	65.71	--	Yes	Yes	Yes	Yes	This work
$Cs_2Na_{0.2}Ag_{0.8}InCl_6$:20%Mn	Perovskites,180	365 nm	>5400 s	32	--	--	--	--	--	1
$Cs_2Na_{0.2}Ag_{0.8}InCl_6$:40%$Yb^{3+}$	Perovskites,180	365 nm	~7200 s	82	--	--	--	--	--	2
$Cs_2NaSc_{1-x}Cl_6$: xTb^{3+}	Perovskites,180	X-ray (50 kV)	12 h	98.2	--	--	--	--	--	3
$CsCdCl_3$:2%Mn	Perovskites,180	254 nm	1480 s	91.4	--	--	--	--	yes	4
$CsCdCl_3$: Mn	Perovskites,180	254 nm	150 s	81.5	--	--	--	--	yes	5
1mol% TMB/PPT	OLPL,250	340 nm	5,000 s	7± 2	--	--	--	--	--	6
1 wt% TMB/PBPO	OLPL,190	365 nm	7 min	3± 1	--	--	--	--	--	7
TPP + BINAP-CuI	OLPL,	370 nm	3 hours	36.9	--	--	--	--	--	8
DAP-Br	OLPL,	450 nm	12 min	--	--	--	--	--	--	9
$Y_3(Al/Ga)_5O_{12}$: $Ce^{3+}/Cr^{3+}/Nd^{3+}$	Phosphors, 1000	410 nm	>1h	--	--	--	--	--	--	10
$ZnGa_2O_4$:Cr^{3+}/Sn^{4+}	Phosphors, 1000	265 nm	7200 s	--	--	--	--	--	--	11

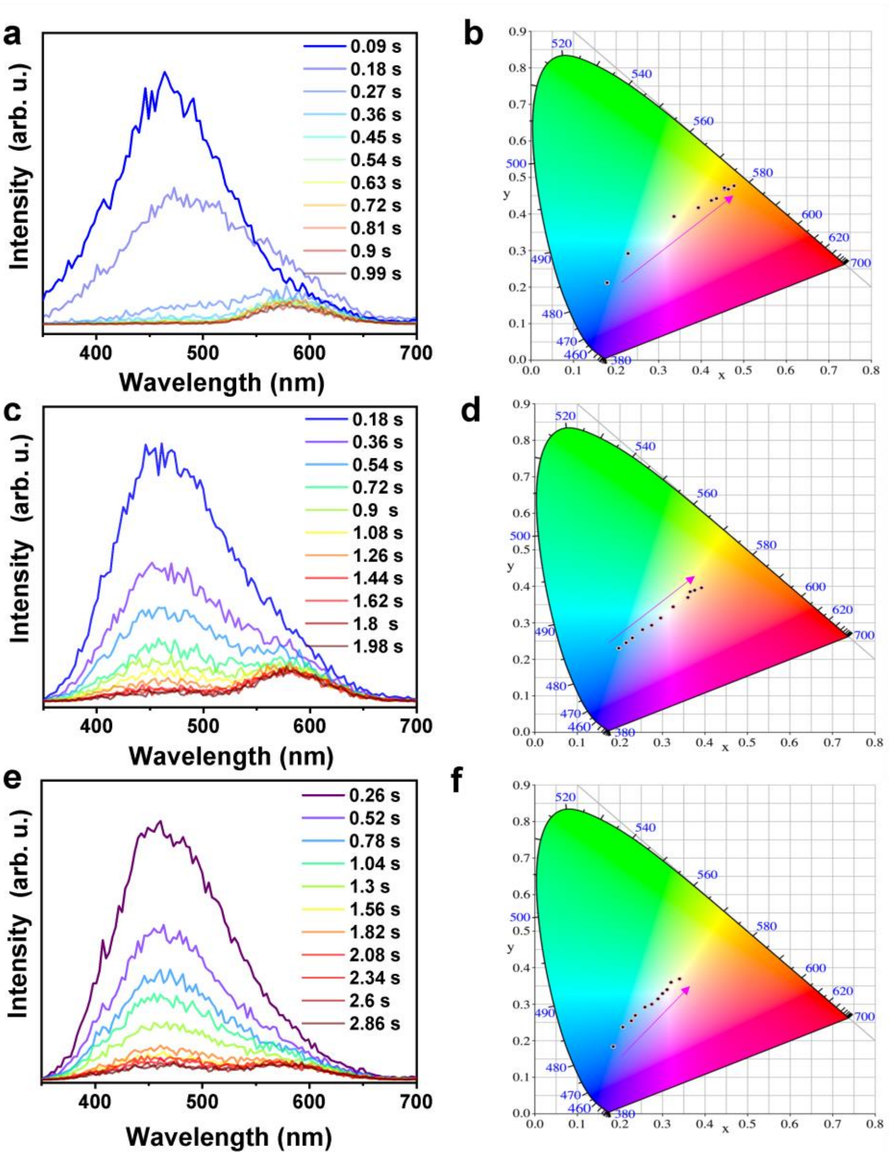

Supplementary Fig. 15 Time-dependent emission spectra and the corresponding CIE of **a-b** $CsCdCl_3$:0.2%Br, **c-d** $CsCdCl_3$:0.5%Br and **e-f** $CsCdCl_3$:0.8%Br.

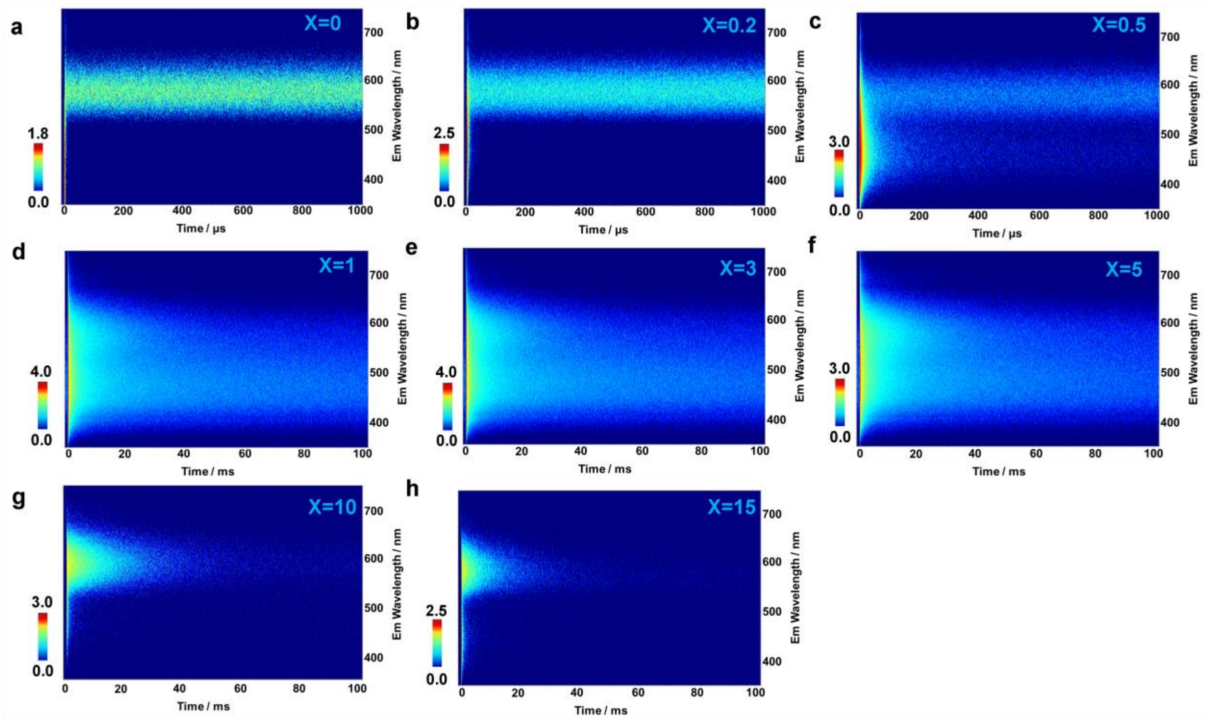

Supplementary Fig. 16 Pseudo color map of time-resolved PL spectra of $CsCdCl_3$:*x*%Br, *x*= **a** 0, **b** 0.2, **c** 0.5, **d** 1, e) 3, **f** 5, **g** 10 and **h** 15 under 254 nm μF900 flash lamp.

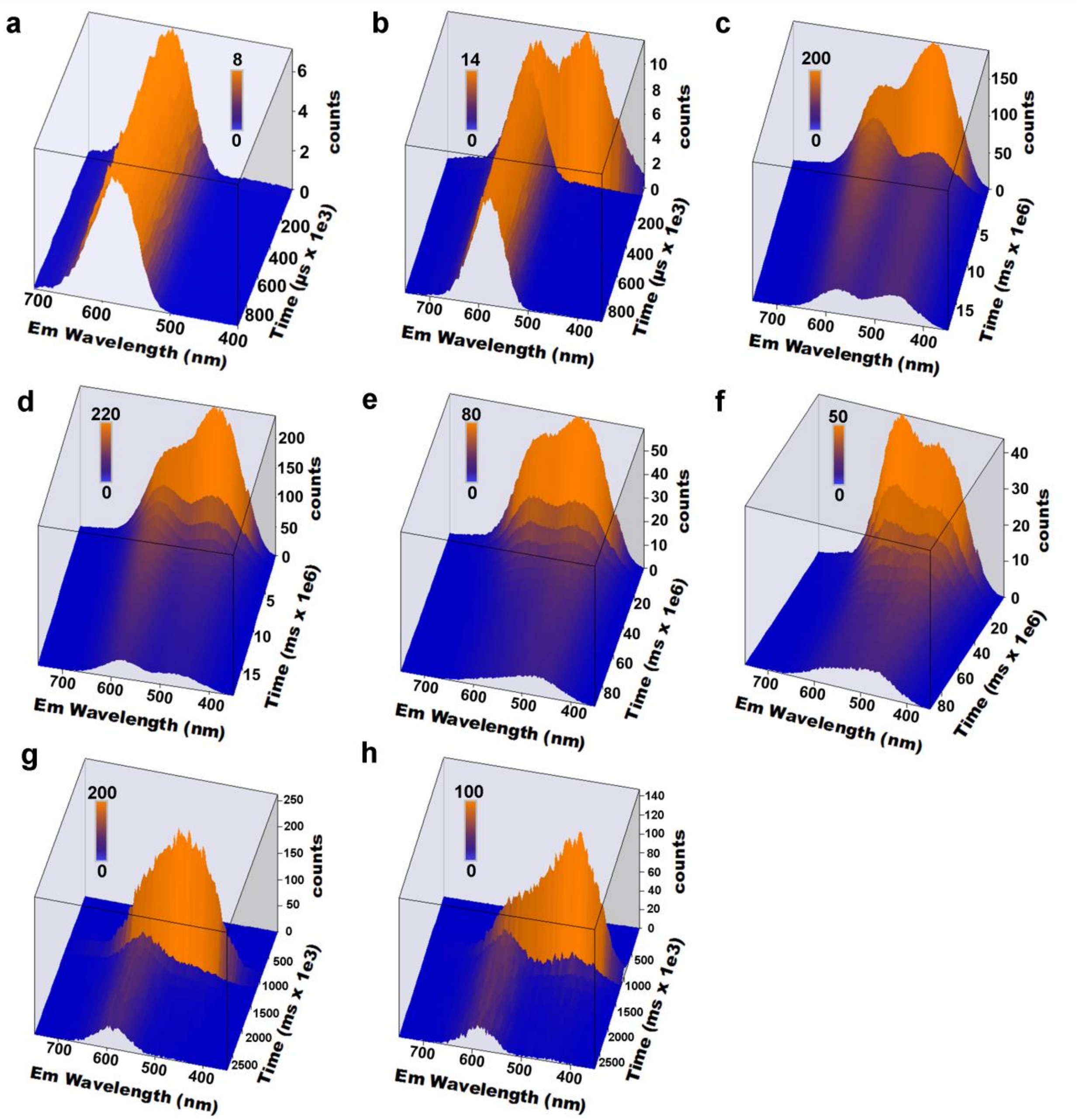

Supplementary Fig. 17 Three-dimensional time-resolved photoluminescence spectra of $CsCdCl_3$:*x*%Br, *x*= **a** 0, **b** 0.2, **c** 0.5, **d** 1, **e** 3, **f** 5, **g** 10 and **h** 15 under 254 nm excitation.

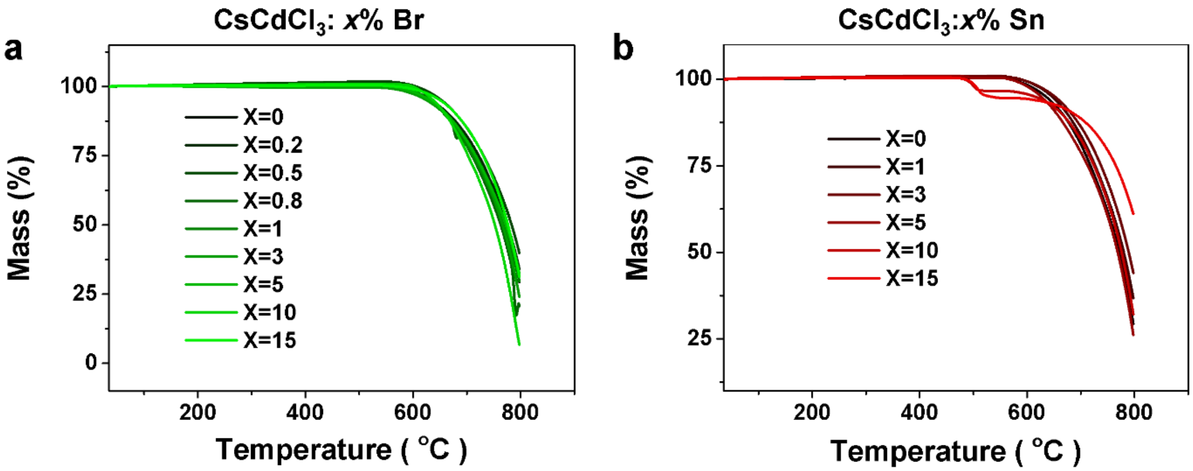

Supplementary Fig. 18 The TGA curves of **a** $CsCdCl_3$:*x*%Br and **b** $CsCdCl_3$:*x*%Sn.

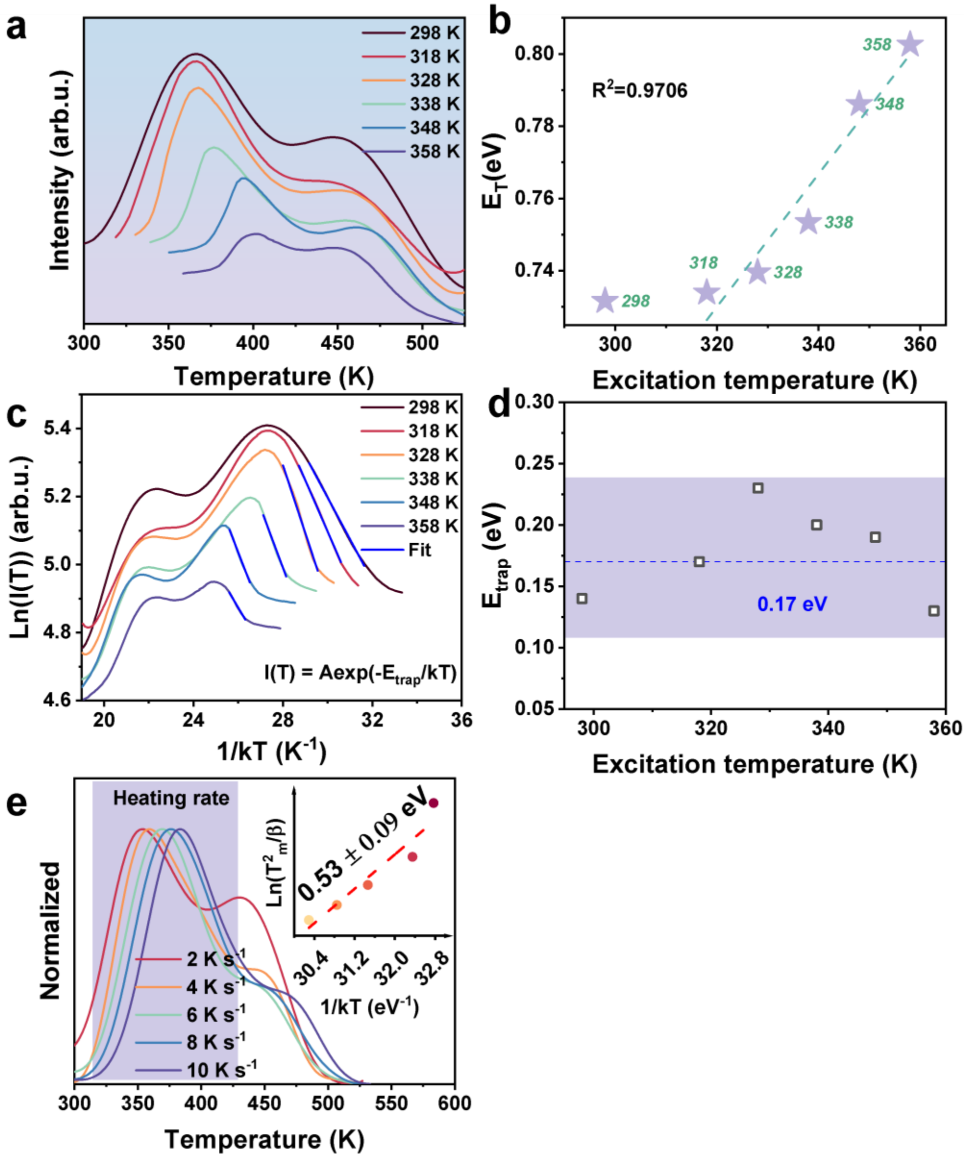

Supplementary Fig. 19 a Results of the partial cleaning ($T_{max} - T_{exc}$) experiments. The TL glow curves of $CsCdCl_3$:0.8%Br were recorded after thermal cleaning at various excitation temperature (T_{exc}). **b** Dependence of E_{trap} on excitation temperature. **c** The dependence of ln(I(T)) on 1/kT, the blue fitting line was determined using the initial rise method. **d** The shallowest trap distribution of the $CsCdCl_3$:0.8%Br. **e** Estimation of trap depth with the Hoogenstraaten method. Hoogenstraaten's peak position characterization is best known and can be expressed as follows:

$$\frac{\beta_h \cdot \varepsilon}{k_B T_m^2} = s \cdot \exp\left(\frac{-\varepsilon}{k_B T_m^2}\right) \qquad \text{S2}$$

where β_h is the heating rate (in K s^{-1}), ε is the trap depth (in eV), k_B is the Boltzmann constant, T_m is the peak temperature in the TL glow curves, and s is the frequency factor (in s^{-1}).

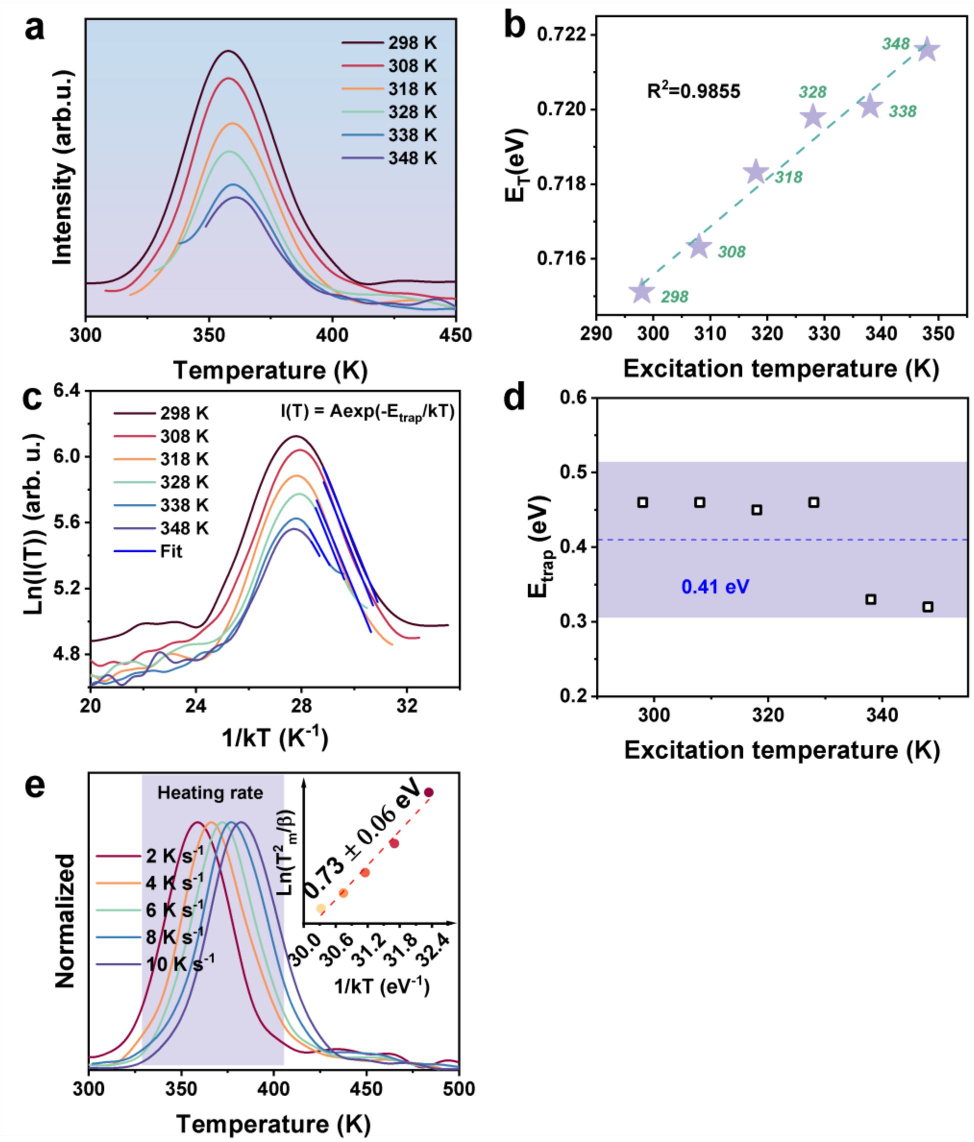

Supplementary Fig. 20 a Results of the partial cleaning ($T_{max} - T_{exc}$) experiments. The TL glow curves of $CsCdCl_3$:10%Br were recorded after thermal cleaning at various excitation temperature (T_{exc}). **b** Dependence of E_{trap} on excitation temperature. **c** The dependence of ln(I(T)) on 1/kT, the blue fitting line was determined using the initial rise method. **d** The shallowest trap distribution of the $CsCdCl_3$:10%Br. **e** Estimation of trap depth with the Hoogenstraaten method.

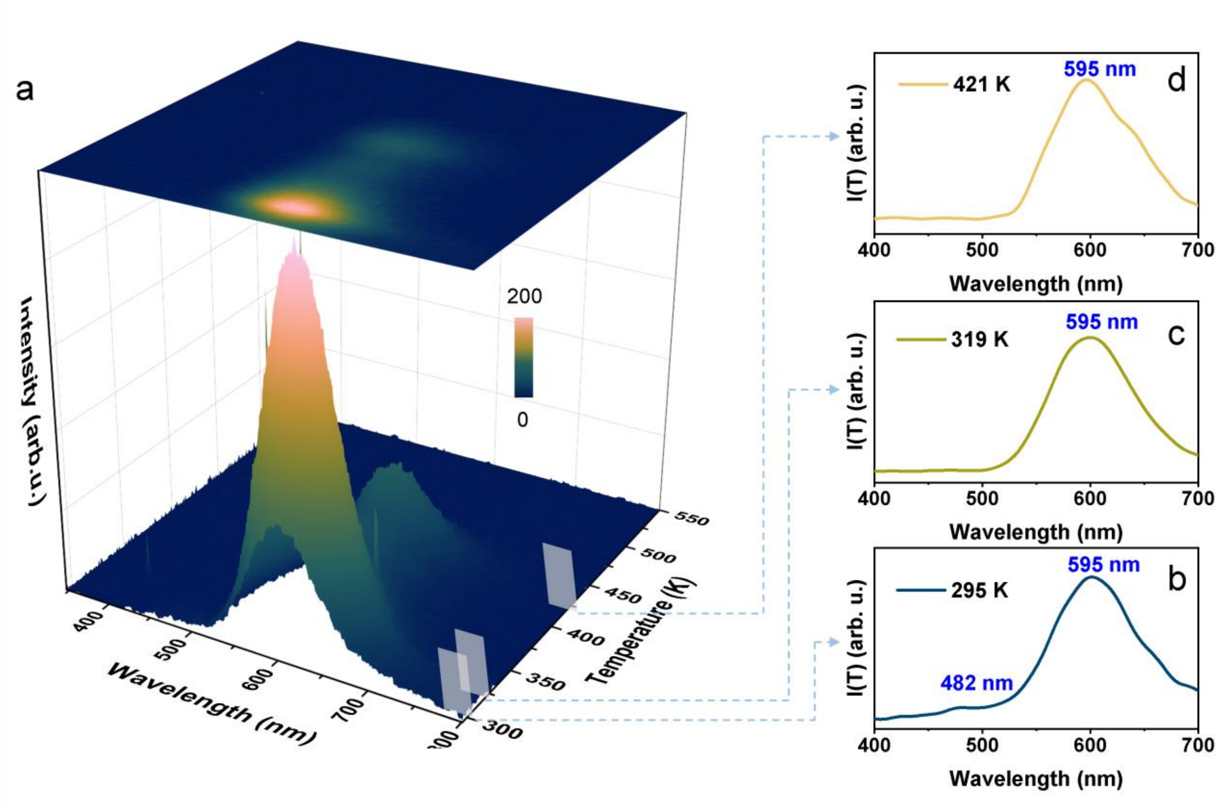

Supplementary Fig. 21 a 3D TL spectra of $CsCdCl_3$:0.8%Br as a function of emission wavelength and temperature after UV (254 nm) illumination for 1 min. Wavelength– resolved TL spectra of $CsCdCl_3$:0.8%Br were monitored at **b** 295K, **c** 319 K and **d** 421 K.

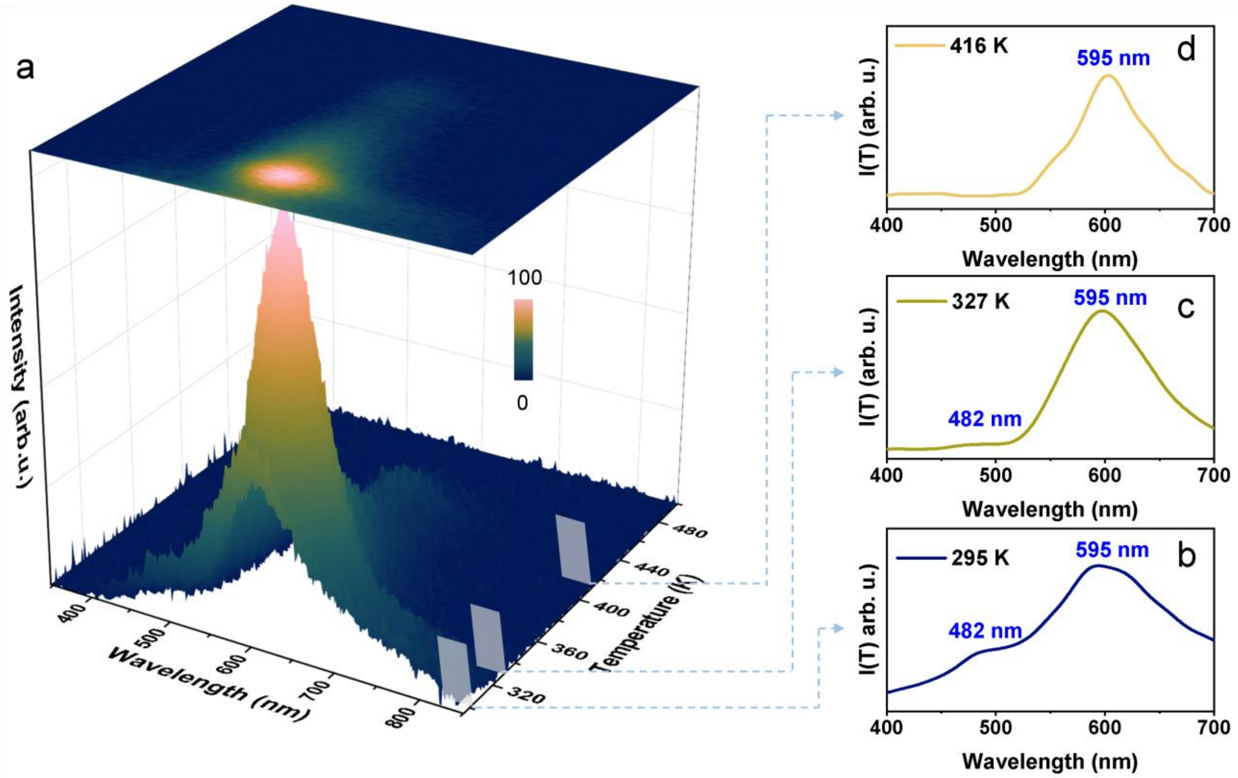

Supplementary Fig. 22 a 3D TL spectra of $CsCdCl_3$:10%Br as a function of emission wavelength and temperature after UV (254 nm) illumination for 1 min. Wavelength– resolved TL spectra of $CsCdCl_3$:10%Br were monitored at **b** 295K, **c** 327 K and **d** 416 K.

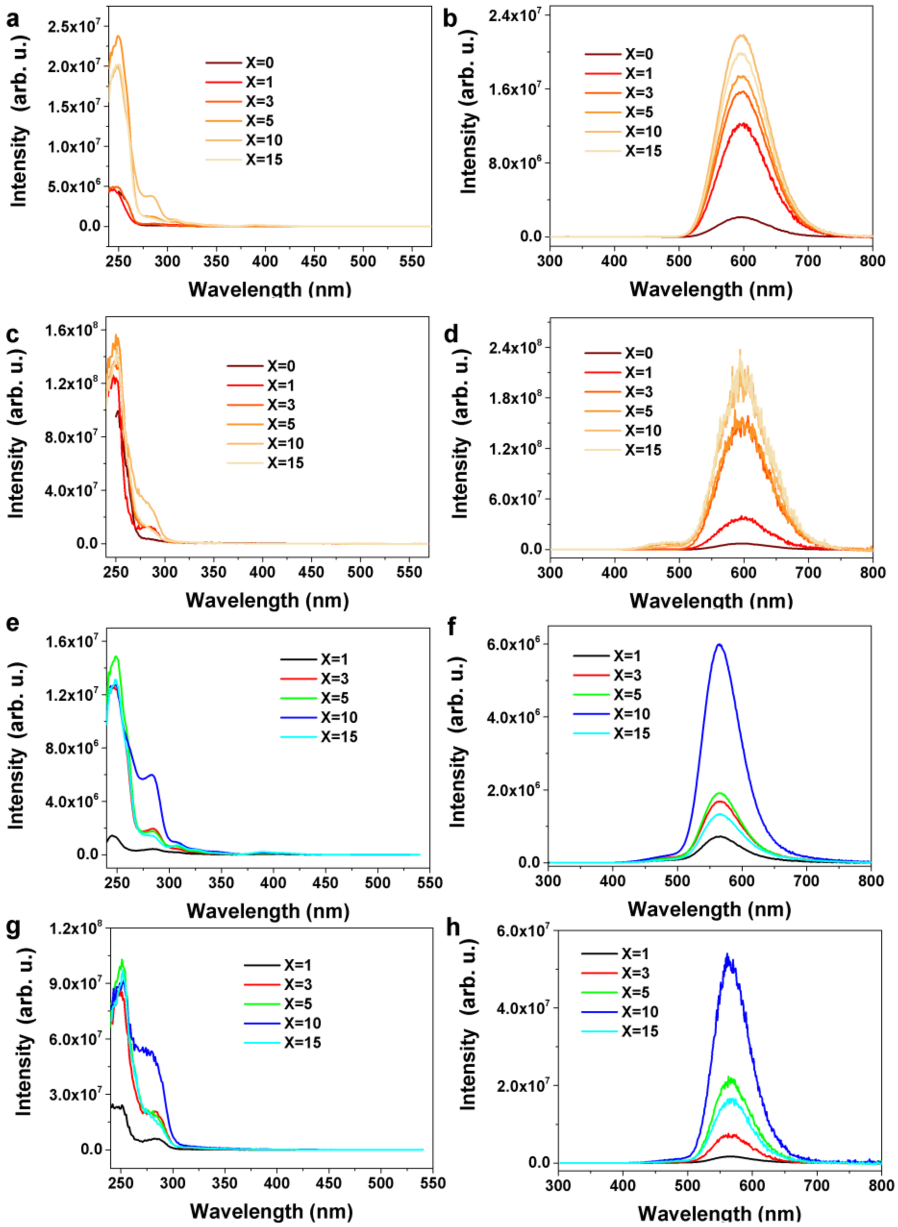

Supplementary Fig. 23. Photoluminescence spectral studies. The PLE spectra of $CsCdCl_3$:*x*%Sn were monitored at 595 nm in **a** prompt and **c** delayed pattern. **b** Prompt and **d** delayed emission (t_d = 1 ms) spectra of $CsCdCl_3$:*x*%Sn under 254 nm excitation. The PLE spectra of $CsCdCl_3$:*x*% Sn were monitored at 565 nm in **e** prompt and **g** delayed pattern. **f** Prompt and **h** delayed emission (t_d = 1 ms) spectra of $CsCdCl_3$:*x*%Sn under 282 nm excitation. *x*=0, 1, 3, 5, 10 and 15.

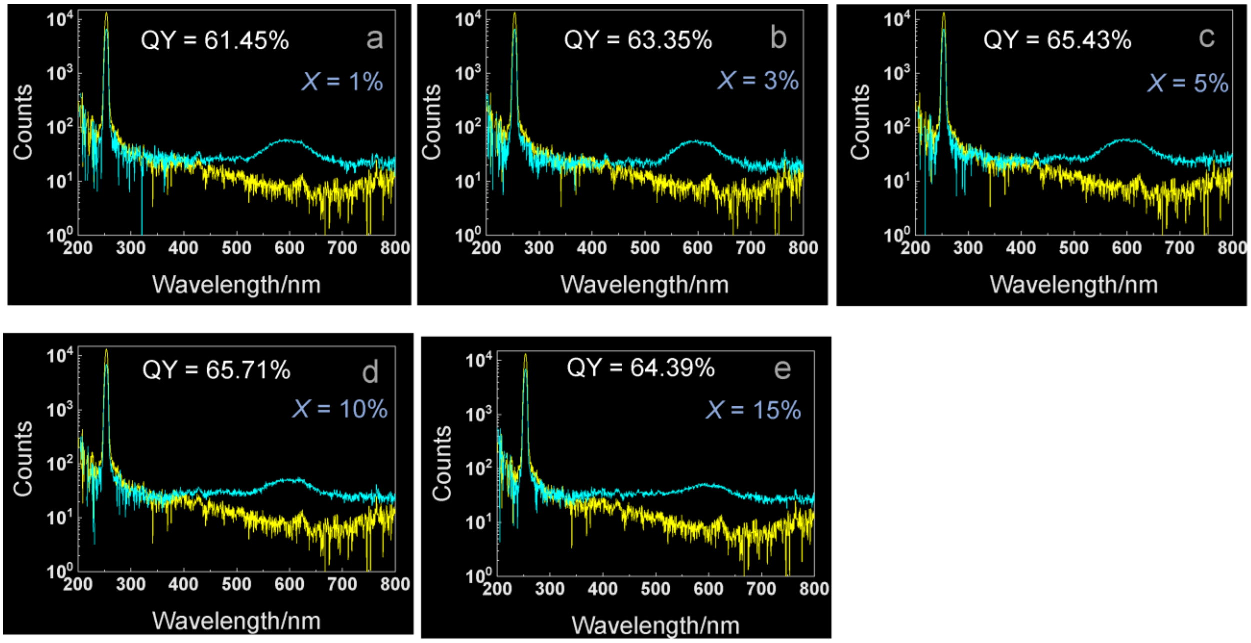

Supplementary Fig. 24 a-e Quantum yields of $CsCdCl_3$:*x*%Sn (x=0, 1, 3, 5, 10, 15).

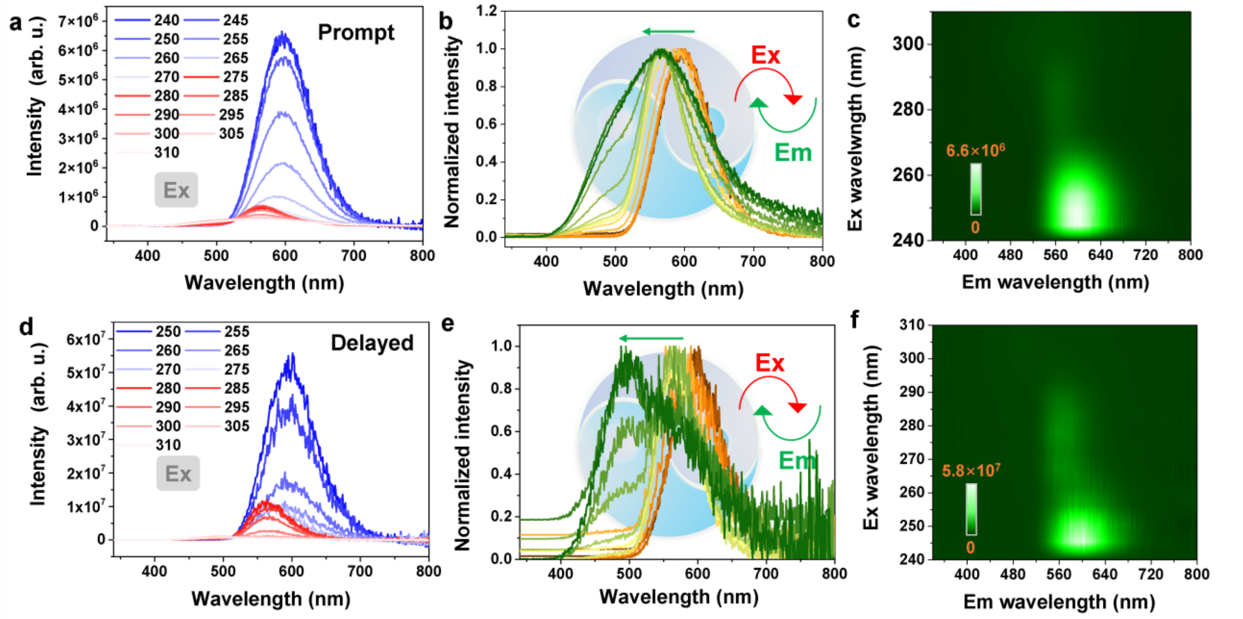

Supplementary Fig. 25. Excitation-wavelength-dependent photoluminescence spectral studies. a Prompt and (d) delayed (t_d = 1 ms) PL spectra for $CsCdCl_3$:1%Sn in irradiation-responsive mode. The normalized **b** prompt and **e** delayed (t_d =1 ms) PL spectra of $CsCdCl_3$:1%Sn exhibit reverse excitation-dependent properties in irradiation-responsive mode at room temperature. **c** Excitation-prompt mapping and **f** excitation-delayed mapping (acquired after 1 ms

of excitation) for $CsCdCl_3$:1%Sn. Note: Due to limited space of the picture, the color gradient of curves in Supp. Fig 25 b,e only represents the change in emission peaks based on excitation-wavelength (the arrows from right to left: 250, 255, 260, 265, 270, 275, 280, 285, 290, 295, 300, 305, and 310 nm, respectively), without any other special meaning.

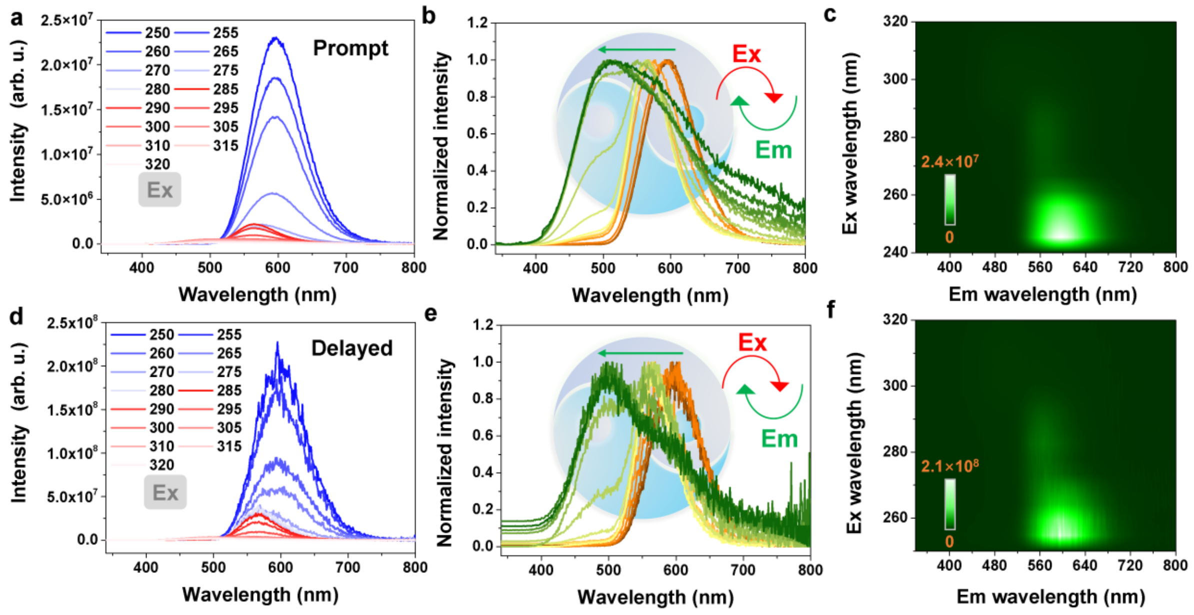

Supplementary Fig. 26. Excitation-wavelength-dependent photoluminescence spectral studies. a Prompt and **d** delayed (t_d = 1 ms) PL spectra for $CsCdCl_3$:3%Sn in irradiation-responsive mode. The normalized **b** prompt and **e** delayed (t_d=1 ms) PL spectra of $CsCdCl_3$:3%Sn exhibit reverse excitation-dependent properties in irradiation-responsive mode at room temperature. **c** Excitation-prompt mapping and **f** excitation-delayed mapping (acquired after 1 ms of excitation) for $CsCdCl_3$:3%Sn. Note: Due to limited space of the picture, the color gradient of curves in Supp. Fig 26 b,e only represents the change in emission peaks based on excitation-wavelength (the arrows from right to left: 250, 255, 260, 265, 270, 275, 280, 285, 290, 295, 300, 305, 310, 315 and 320 nm, respectively), without any other special meaning.

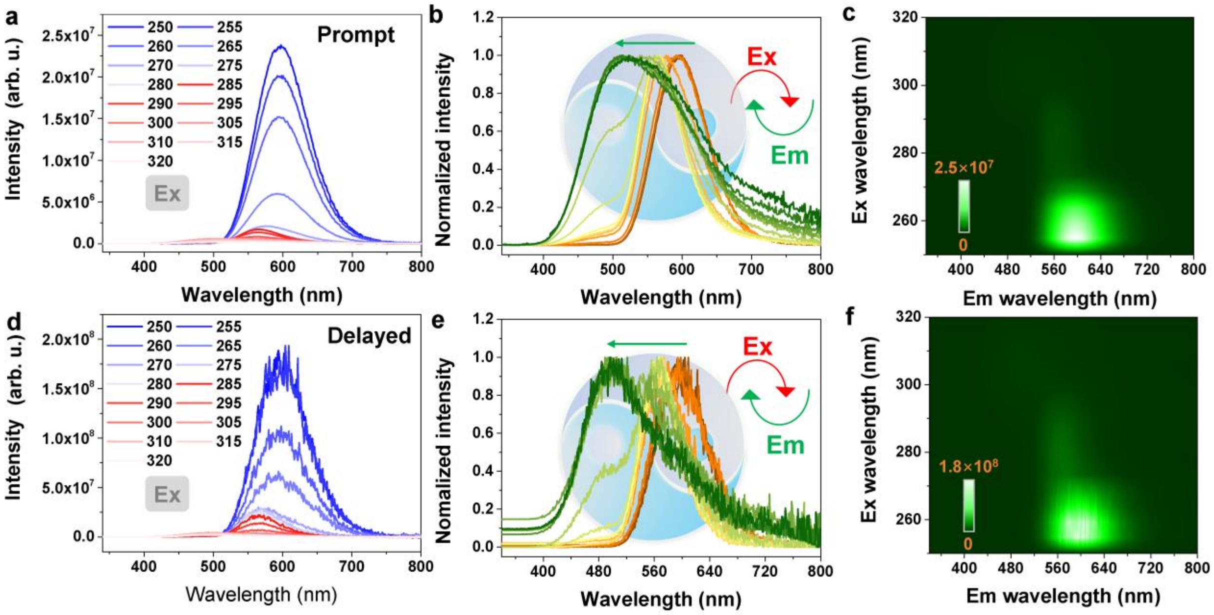

Supplementary Fig. 27. Excitation-wavelength-dependent photoluminescence spectral studies. a Prompt and **d** delayed (t_d = 1 ms) PL spectra for $CsCdCl_3$:5%Sn in irradiation-responsive mode. The normalized **b** prompt and **e** delayed (t_d=1 ms) PL spectra of $CsCdCl_3$:5%Sn exhibit reverse excitation-dependent properties in irradiation-responsive mode at room temperature. **c** Excitation-prompt mapping and **f** excitation-delayed mapping (acquired after 1 ms of excitation) for $CsCdCl_3$:5%Sn. Note: Due to limited space of the picture, the color gradient of curves in Supp. Fig 27 b,e only represents the change in emission peaks based on excitation-wavelength (the arrows from right to left: 250, 255, 260, 265, 270, 275, 280, 285, 290, 295, 300, 305, 310, 315 and 320 nm, respectively), without any other special meaning.

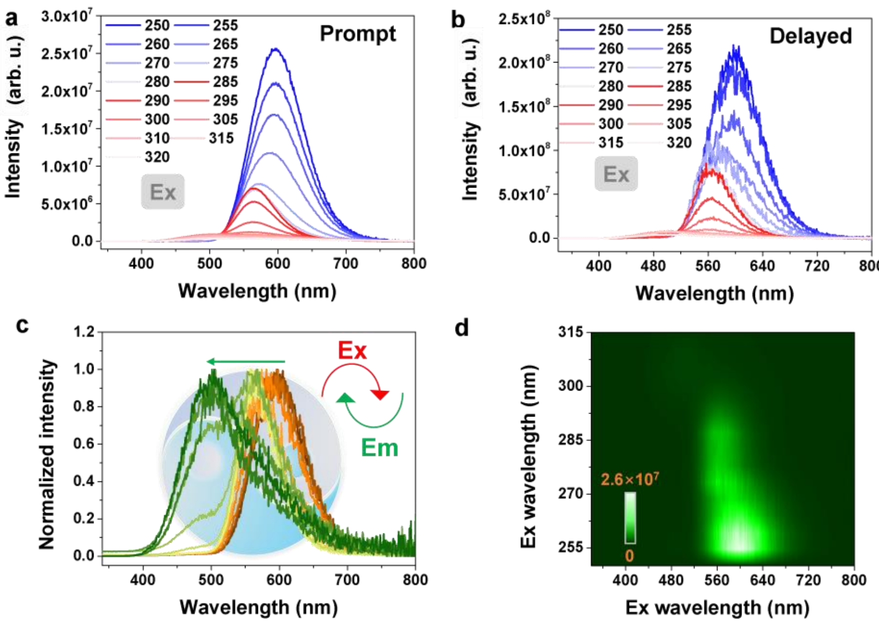

Supplementary Fig. 28. Excitation-wavelength-dependent photoluminescence spectral studies. a Prompt and **b** delayed (t_d = 1 ms) PL spectra for $CsCdCl_3$:10%Sn in irradiation-responsive mode. **c** The normalized delayed (t_d =1 ms) PL spectra of $CsCdCl_3$:10%Sn exhibit reverse excitation-dependent properties in irradiation-responsive mode at room temperature. **d** excitation-delayed mapping (acquired after 1 ms of excitation) for $CsCdCl_3$:10%Sn. Note: Due to limited space of the picture, the color gradient of curves in Supp. Fig 28 c only represents the change in emission peaks based on excitation-wavelength (the arrows from right to left: 250, 255, 260, 265, 270, 275, 280, 285, 290, 295, 300, 305, 310, 315 and 320 nm, respectively), without any other special meaning.

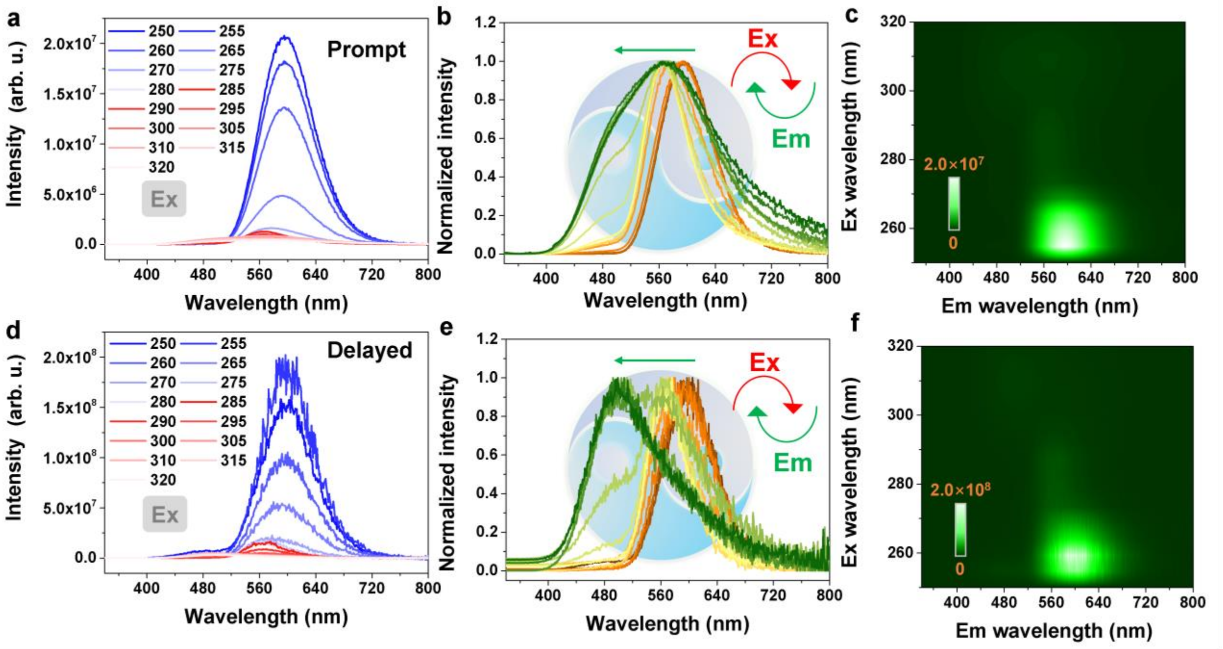

Supplementary Fig. 29. Excitation-wavelength-dependent photoluminescence spectral studies. a Prompt and **d** delayed (t_d = 1 ms) PL spectra for $CsCdCl_3$:15%Sn in irradiation-responsive mode. The normalized **b** prompt and **e** delayed (t_d=1 ms) PL spectra of $CsCdCl_3$:15%Sn exhibit reverse excitation-dependent properties in irradiation-responsive mode at room temperature. **c** Excitation-prompt mapping and **f** excitation-delayed mapping (acquired after 1 ms of excitation) for $CsCdCl_3$:15%Sn. Note: Due to limited space of the picture, the color gradient of curves in Supp. Fig 29 b,e only represents the change in emission peaks based on excitation-wavelength (the arrows from right to left: 250, 255, 260, 265, 270, 275, 280, 285, 290, 295, 300, 305, 310, 315 and 320 nm, respectively), without any other special meaning.

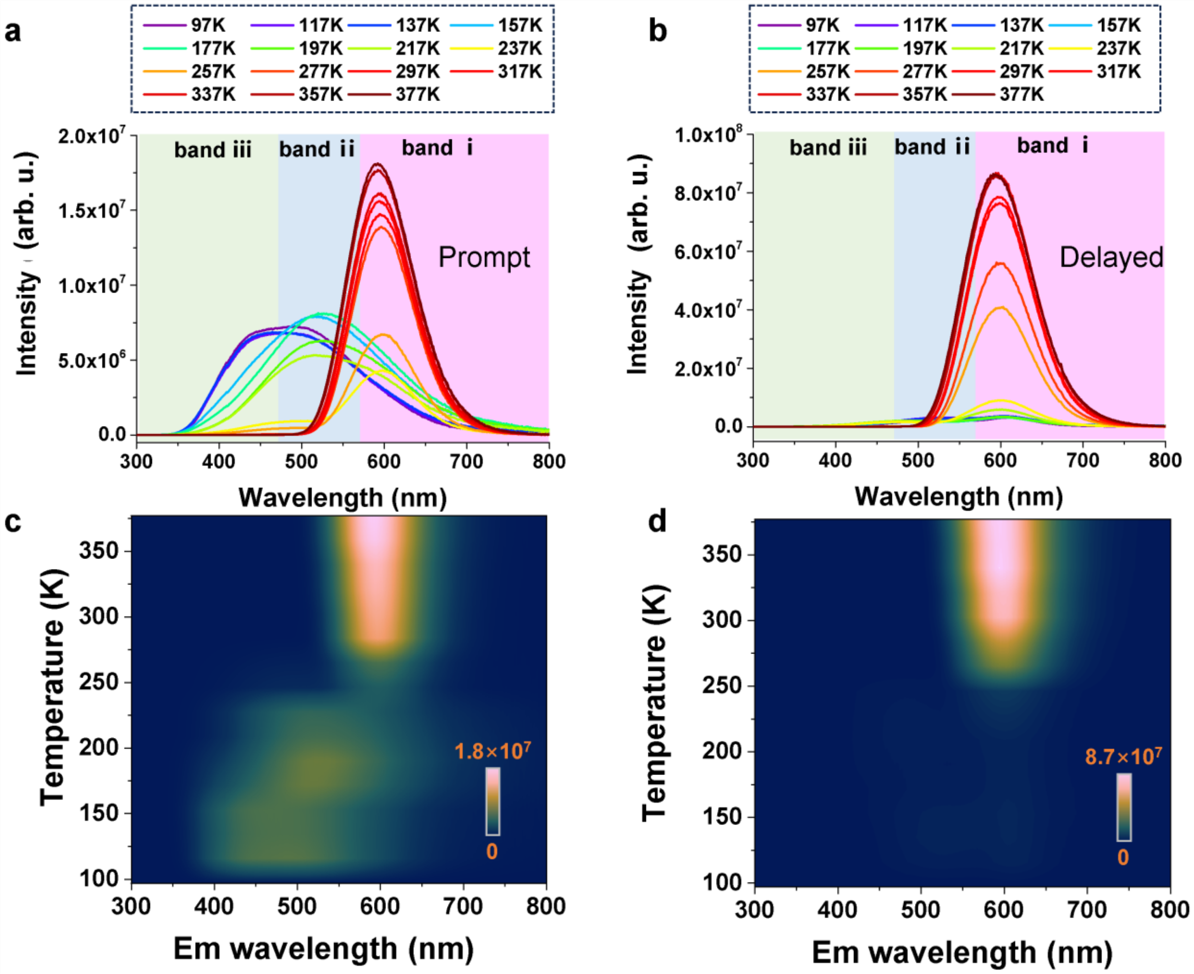

Supplementary Fig. 30. Temperature-dependent photoluminescence spectral studies. The PL spectra of $CsCdCl_3$:3%Sn at different temperatures ranging from 97 to 377K based on **a** prompt and **b** delayed (t_d = 1 ms) patterns under 254 nm excitation. The Pseudo color map of PL spectra of $CsCdCl_3$:3%Sn at different temperatures based on **c** prompt and **d** delayed (t_d = 1 ms) patterns under 254 nm excitation.

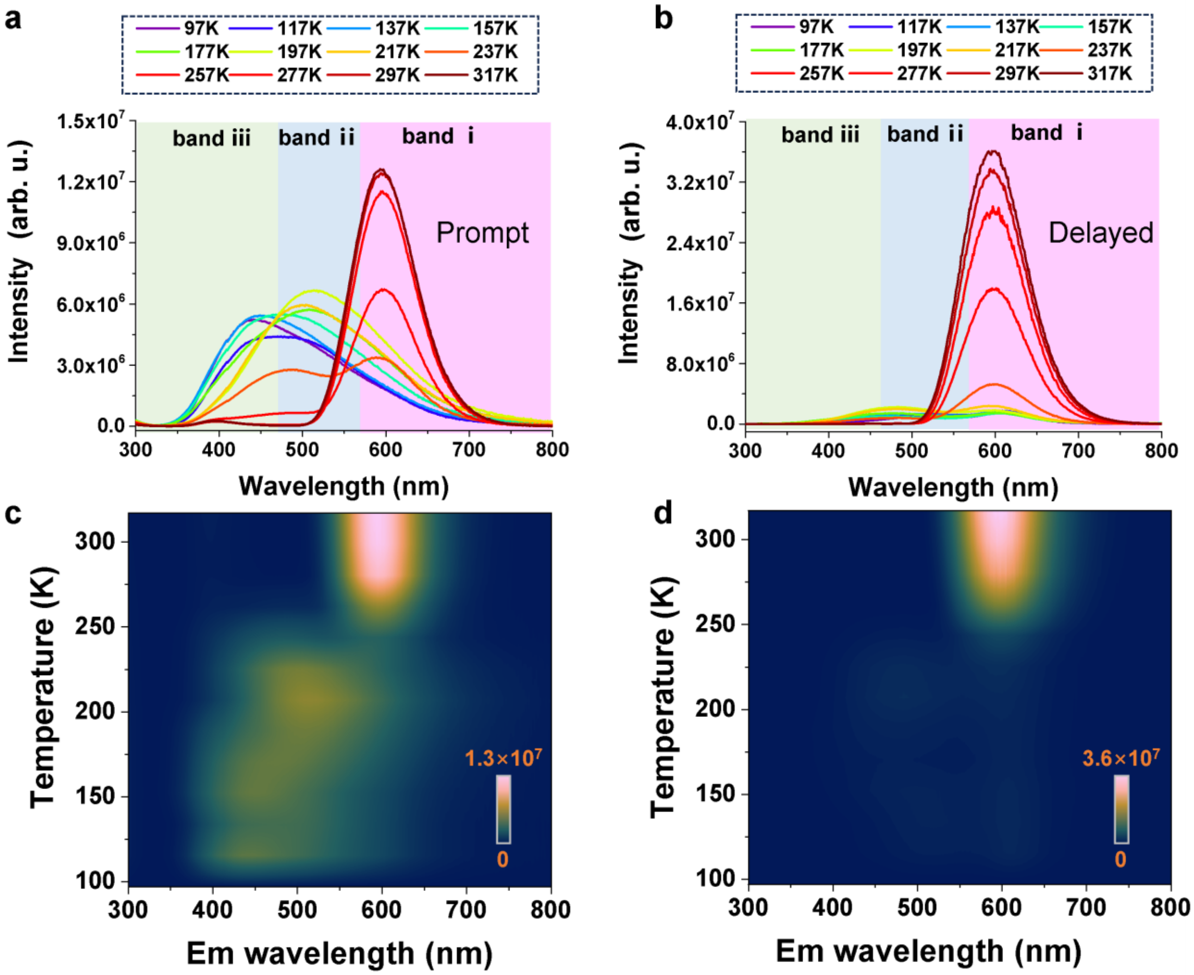

Supplementary Fig. 31. Temperature-dependent photoluminescence spectral studies. The PL spectra of $CsCdCl_3$:10%Sn at different temperatures ranging from 97 to 377 K based on **a** prompt and **b** delayed (t_d = 1 ms) patterns under 254 nm excitation. The Pseudo color map of PL spectra of $CsCdCl_3$:3%Sn at different temperatures based on **c** prompt and **d** delayed (t_d = 1 ms) patterns under 254 nm excitation.

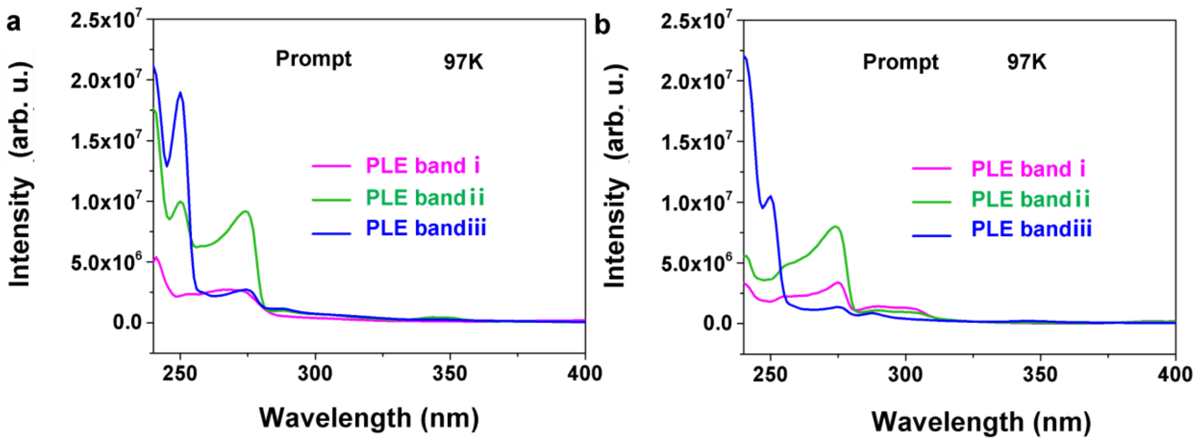

Supplementary Fig. 32 The PLE spectra of **a** $CsCdCl_3$:3%Sn and **b** $CsCdCl_3$:10%Sn were monitored at band i, band ii and band iii in prompt pattern.

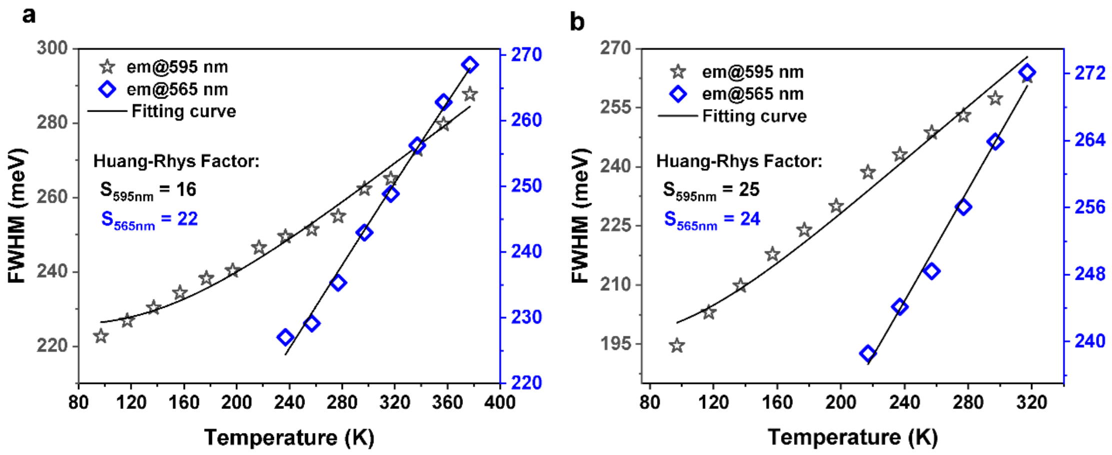

Supplementary Fig. 33. FWHM of **a** $CsCdCl_3$:3%Sn and **b** $CsCdCl_3$:10%Sn at 595 nm and 565 nm versus temperature form the delayed spectra of Supplementary Fig. 27-28, respectively.

The electron-phonon coupling effect of Sn-doped $CsCdCl_3$ was discussed by fitting FWHM versus temperature according to Equation S1.

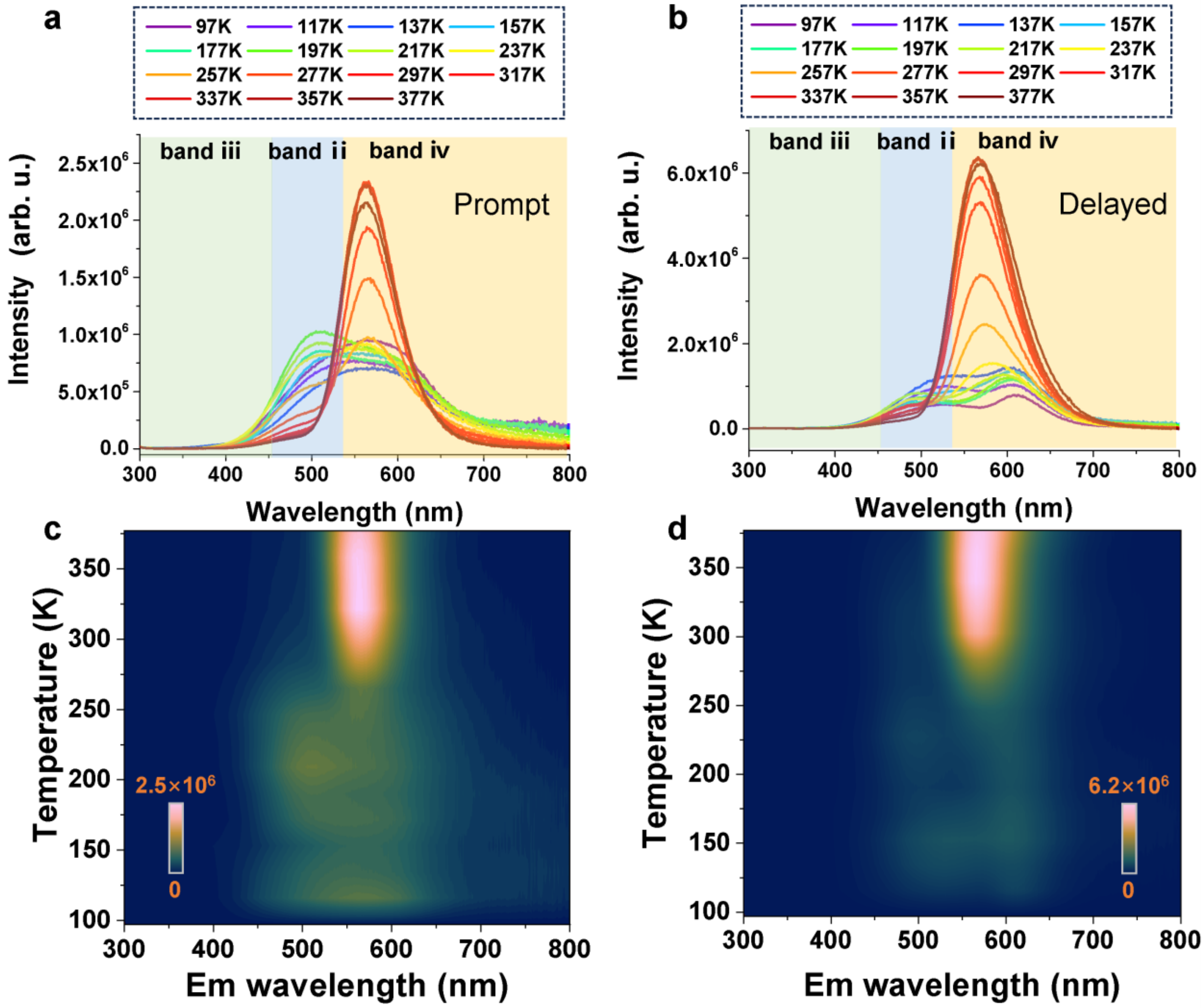

Supplementary Fig. 34. Temperature-dependent photoluminescence spectral studies. The PL spectra of $CsCdCl_3$:3%Sn at different temperatures ranging from 97 to 377 K based on **a** prompt and **b** delayed (t_d = 1 ms) patterns under 282 nm excitation. The Pseudo color map of PL spectra of $CsCdCl_3$:3%Sn at different temperatures based on **c** prompt and **d** delayed (t_d = 1 ms) patterns under 282 nm excitation.

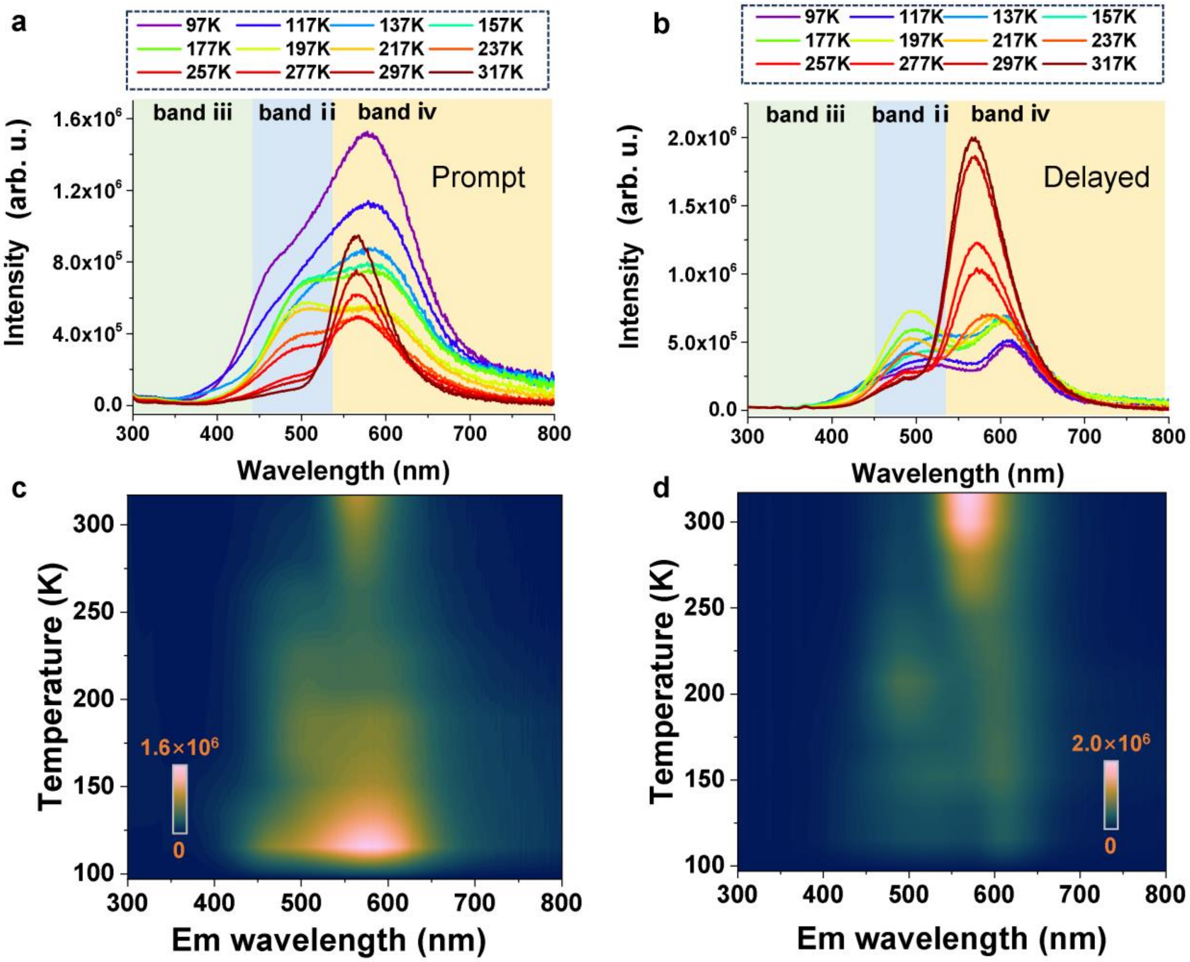

Supplementary Fig. 35. Temperature-dependent photoluminescence spectral studies. The PL spectra of $CsCdCl_3$:10%Sn at different temperatures ranging from 97 to 377 K based on **a** prompt and **b** delayed (t_d = 1 ms) patterns under 283 nm excitation. The Pseudo color map of PL spectra of $CsCdCl_3$:10%Sn at different temperatures based on **c** prompt and **d** delayed (t_d = 1 ms) patterns under 282 nm excitation.

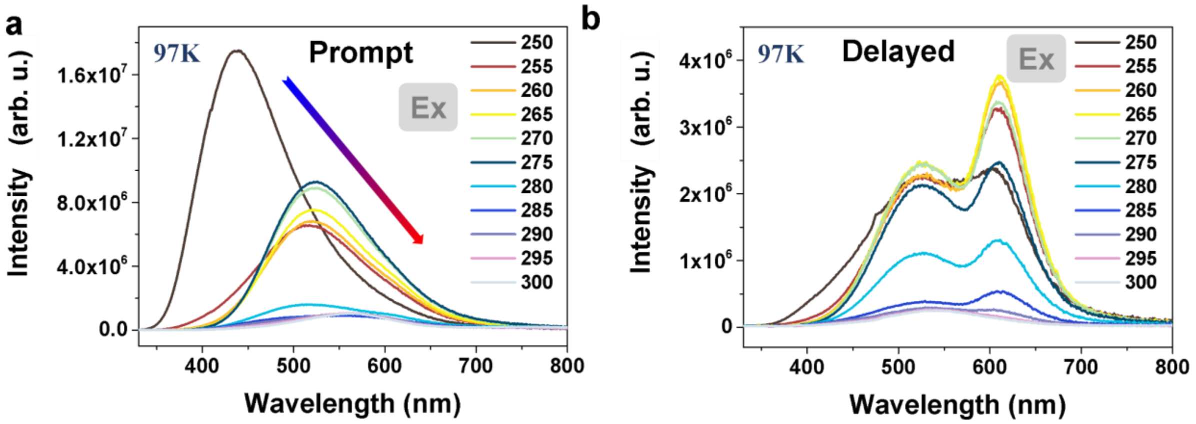

Supplementary Fig. 36. Excitation-wavelength-dependent photoluminescence spectral studies. a Prompt and **b** delayed (t_d = 1 ms) PL spectra for $CsCdCl_3$:3%Sn in irradiation-responsive mode at 97K.

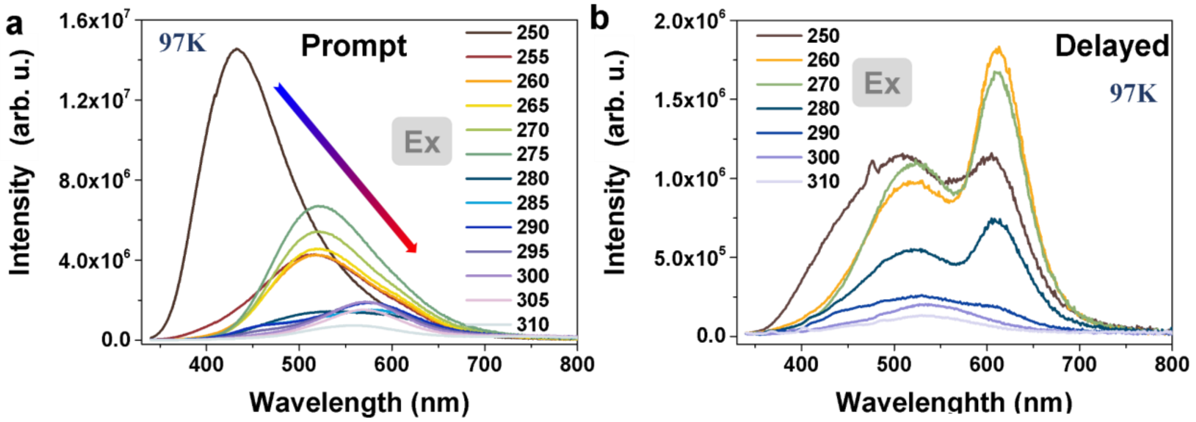

Supplementary Fig. 37. Excitation-wavelength-dependent photoluminescence spectral studies. a Prompt and **b** delayed (t_d = 1 ms) PL spectra for $CsCdCl_3$:10%Sn in irradiation-responsive mode at 97K.

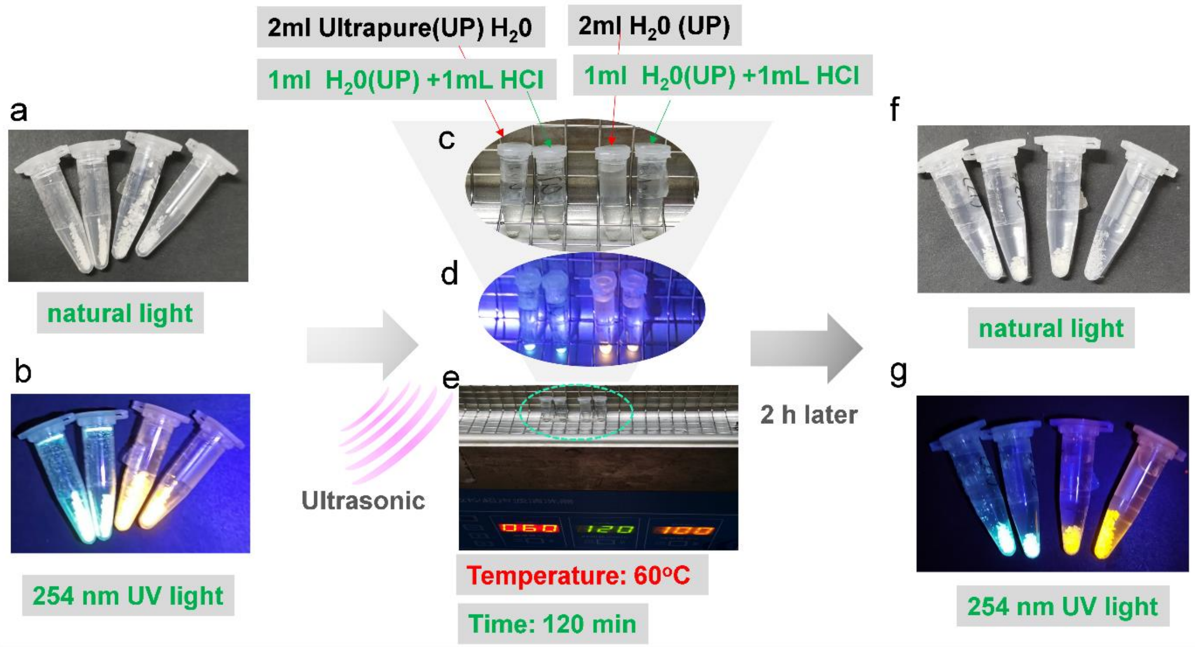

Supplementary Fig. 38. a-g The optical phenomenon of $CsCdCl_3$:5%Br and $CsCdCl_3$:10%Sn were observed in ultra-pure H_2O and HCl (v:v=1:1) solution by ultrasonic treatment at 60 ^oC for two hours, respectively. Note: Supplementary Fig. 36 a, the centrifuge tubes from left to right are $CsCdCl_3$:5%Br, $CsCdCl_3$:5%Br, $CsCdCl_3$:10%Sn and $CsCdCl_3$:10%Sn, respectively.

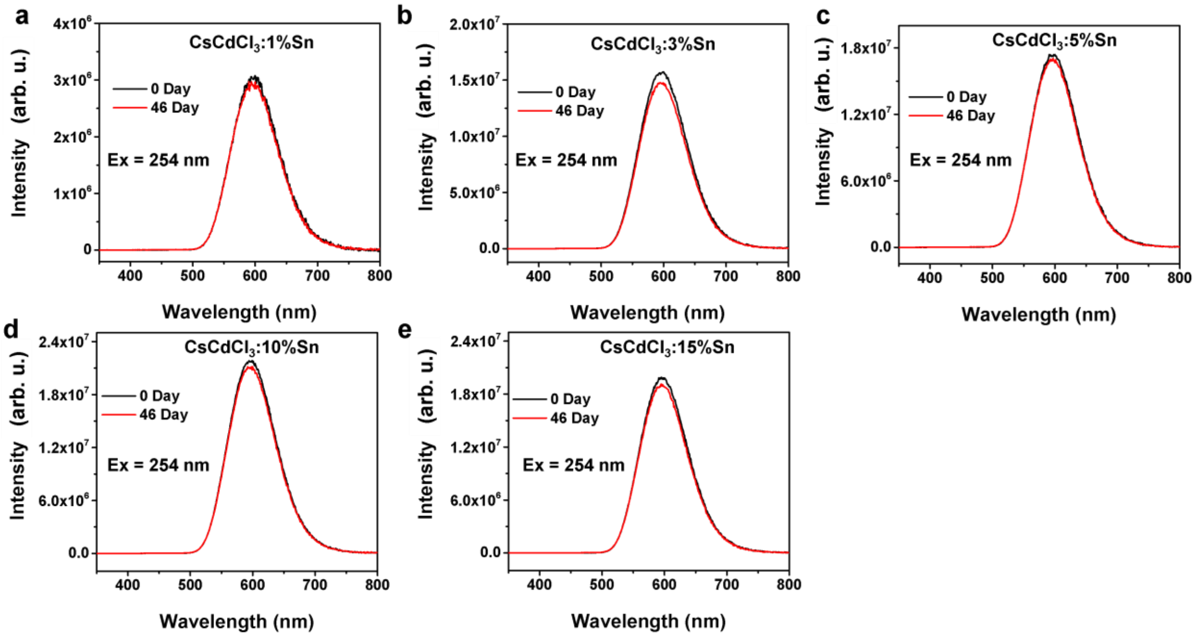

Supplementary Fig. 39 a-e PL spectra of 0 day and 46 days stored at room temprature of $CsCdCl_3$:x%Sn (x=0, 1, 3, 5, 10, 15) (λ_{ex} = 254 nm).

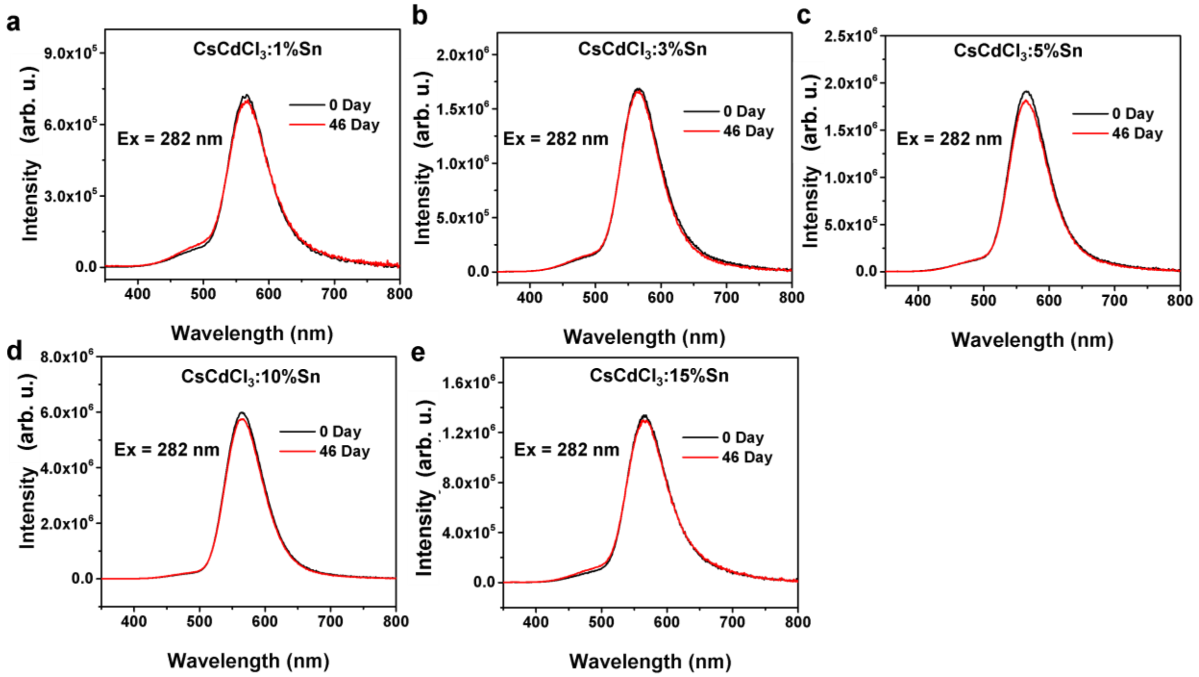

Supplementary Fig. 40 a-e PL spectra of 0 day and 46 days stored at room temprature of $CsCdCl_3$:x%Sn (x=0, 1, 3, 5, 10, 15) (λ_{ex} = 282 nm).

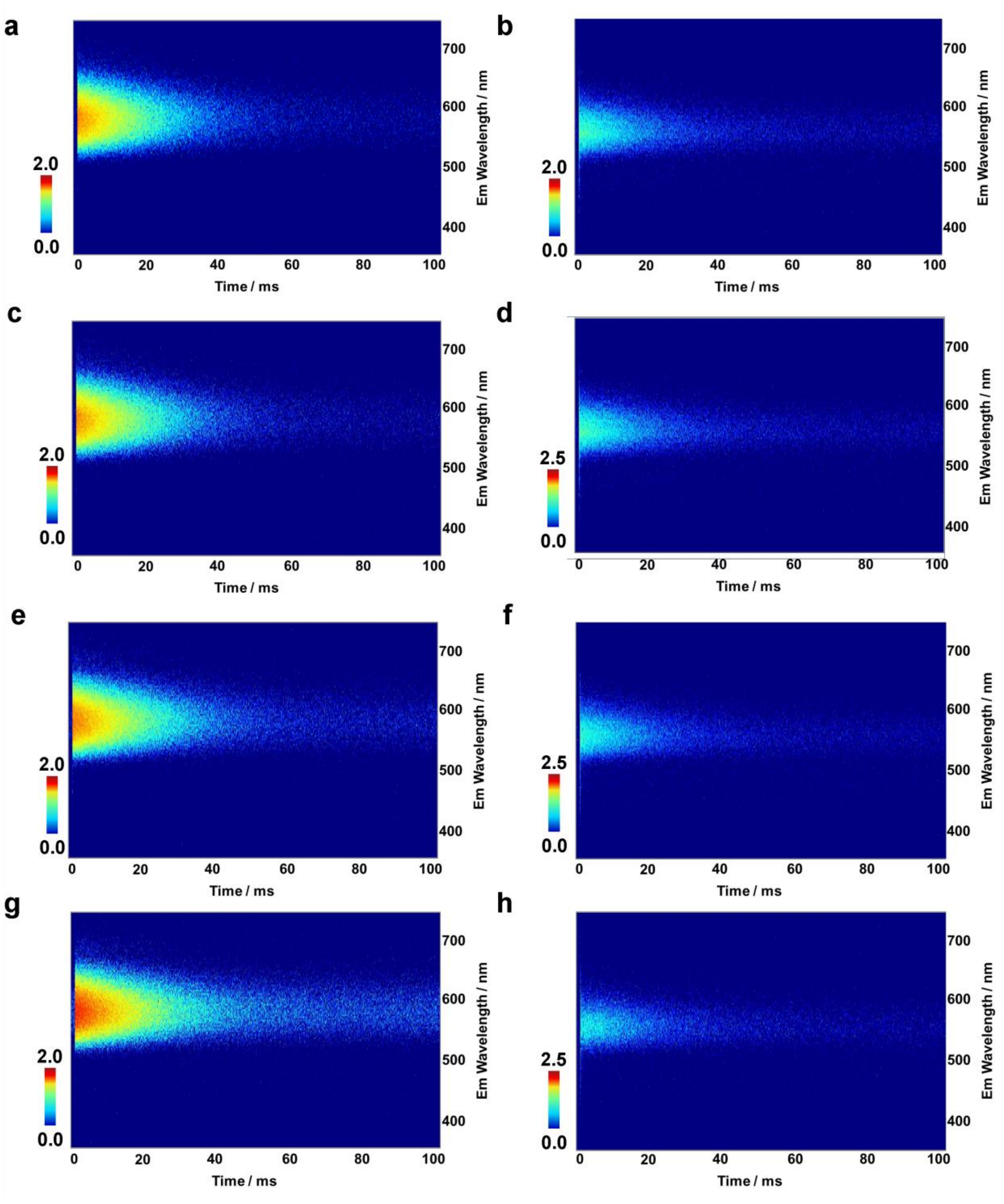

Supplementary Fig. 41 Pseudo color map of time-resolved PL spectra of $CsCdCl_3$:*x*%Sn, x= **a** 1, **c** 3, **e** 5 and **g** 15 under 254 nm μF900 flash lamp, and x= **b** 1, **d** 3, **f** 5 and **h** 15 under 282 nm μF900 flash lamp.

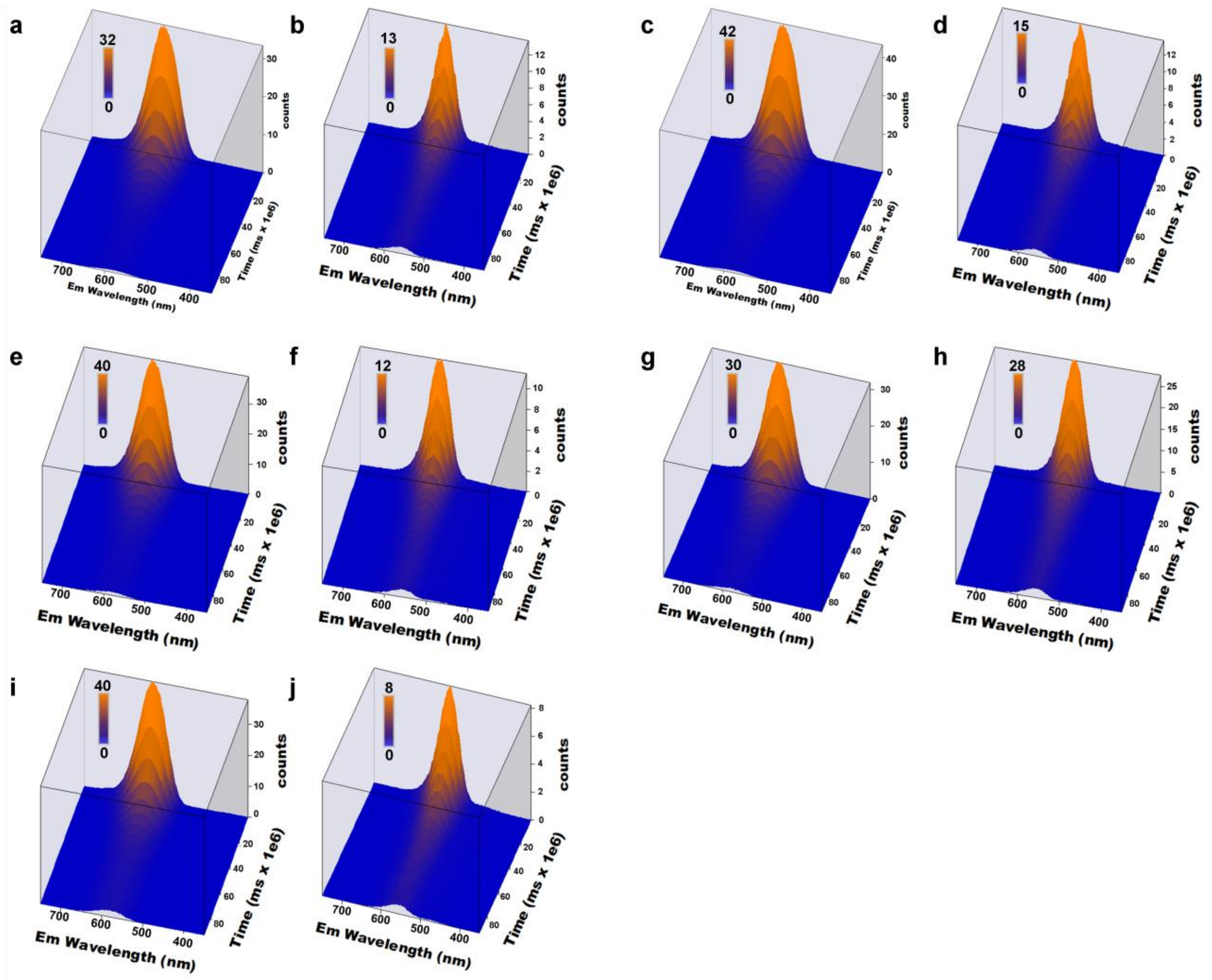

Supplementary Fig. 42 Three-dimensional time-resolved photoluminescence spectra of $CsCdCl_3$:*x*%Sn, x= **a** 1, **c** 3, **e** 5, **g** 10 and **i** 15 under 254 nm excitation, and x= **b** 1, **d** 3, **f** 5 and **h** 10 and **j** 15 under 282 nm excitation.

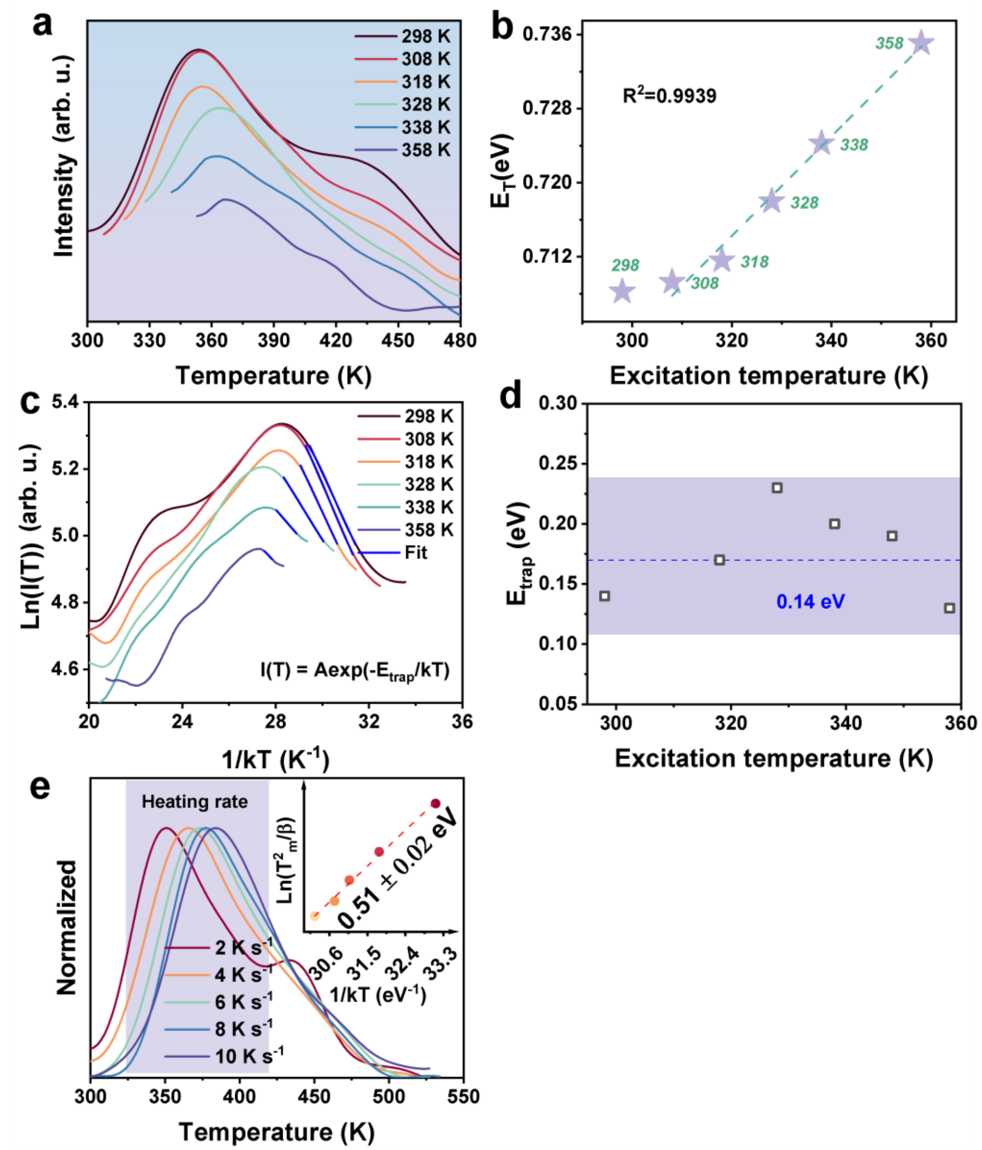

Supplementary Fig. 43 (**a**) Results of the partial cleaning ($T_{max} – T_{exc}$) experiments. The TL glow curves of $CsCdCl_3$:10%Sn were recorded after thermal cleaning at various excitation temperature (T_{exc}). **b** Dependence of E_{trap} on excitation temperature. **c** The dependence of ln(I(T)) on 1/kT, the blue fitting line was determined using the initial rise method. **d** The shallowest trap distribution of the $CsCdCl_3$:10%Sn. **e** Estimation of trap depth with the Hoogenstraaten method.

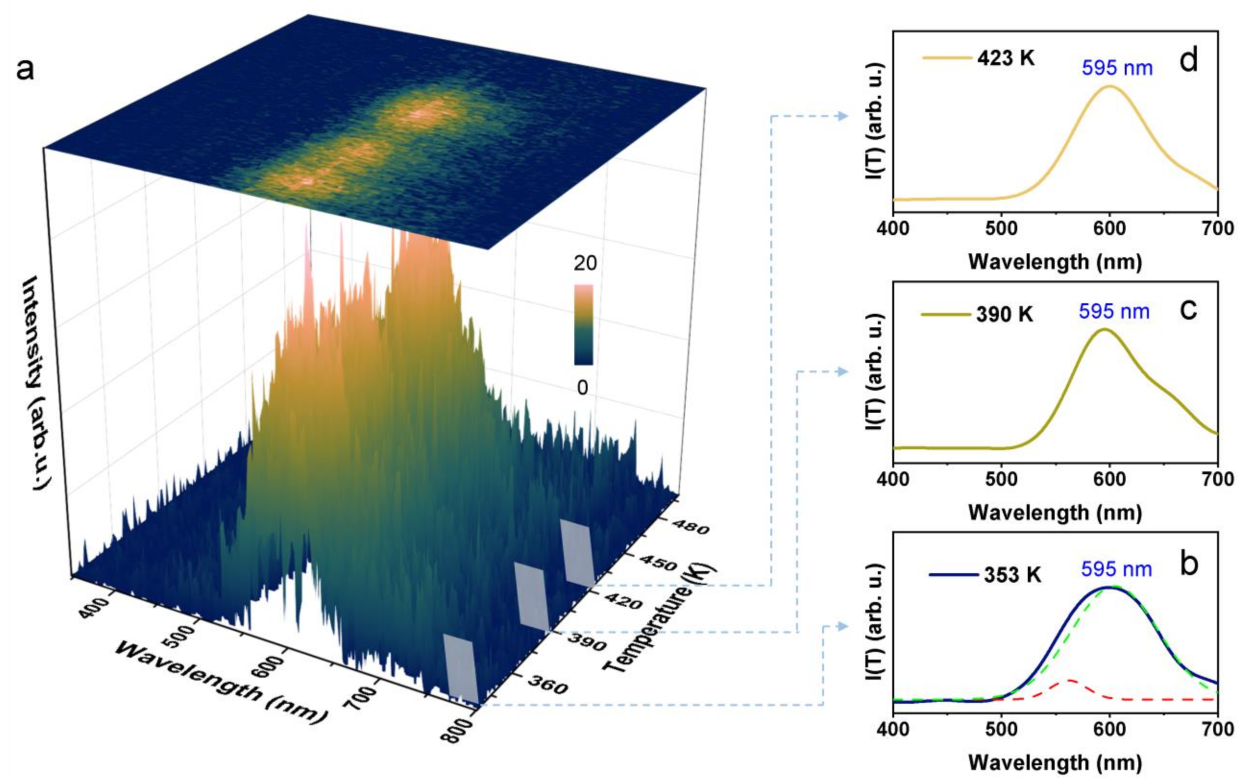

Supplementary Fig. 44 a 3D TL spectra of $CsCdCl_3$:10%Sn as a function of emission wavelength and temperature after UV (254 nm) illumination for 1 min. Wavelength– resolved TL spectra of $CsCdCl_3$:10%Sn were monitored at **b** 353 K, **c** 390 K and **d** 423 K.

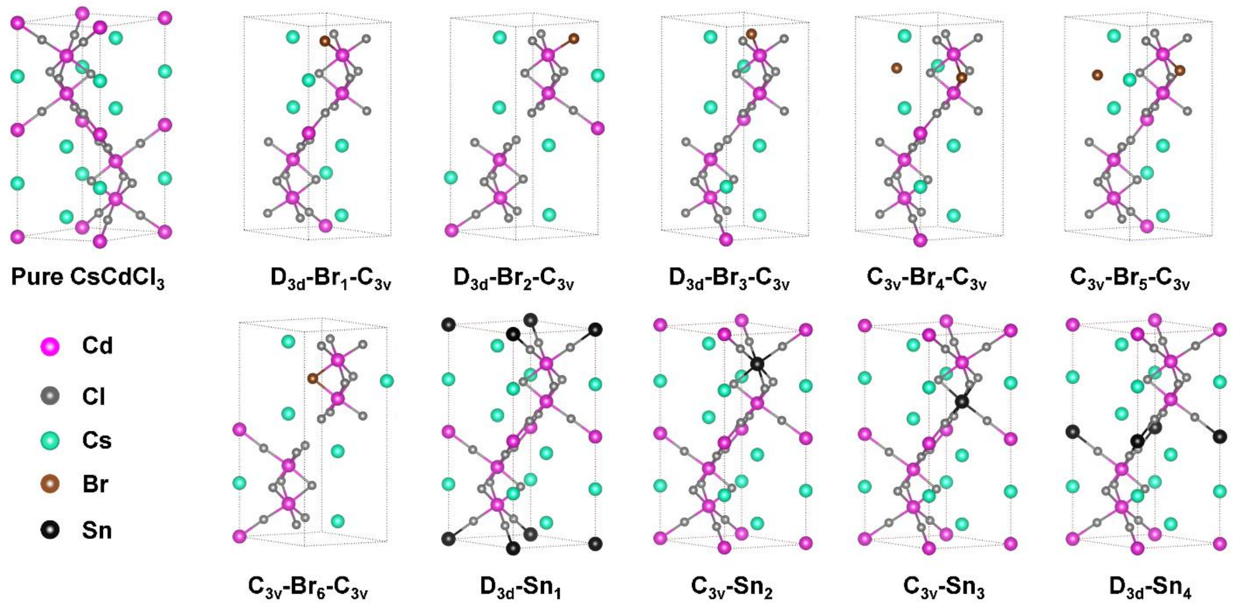

Supplementary Fig. 45. The structural optimization model presents potential doping sites for Br^- ion or Sn^{2+} ion within the hexagonal crystal structure of $CsCdCl_3$.

Supplementary Table 6 Lattice constants of Br^- or Sn^{2+} -doped $CdCdCl_3$ perovskites

Models	a(Å)	b(Å)	c(Å)	V(Å^3)	α^o	β^o	γ^o
D_{3d}-Br_1-C_{3v}	7.5694	7.5996	18.8776	941.6613	89.5989	90	119.8684
D_{3d}-Br_2-C_{3v}	7.5996	7.5996	18.8776	941.6632	90.4010	89.5990	120.2628
D_{3d}-Br_3-C_{3v}	7.5996	7.5694	18.8771	941.6637	90	90.4018	119.8687
C_{3v}-Br_4-C_{3v}	7.5758	7.5749	18.8678	937.7418	90	90	119.9958
C_{3v} -Br_5-C_{3v}	7.5748	7.5757	18.8675	937.6839	90	90	119.9958
C_{3v}-Br_6-C_{3v}	7.5758	7.5758	18.8671	937.6753	90	90	120.0093
D_{3d}-Sn_1	7.6218	7.6218	19.0540	958.6043	90	90	120
C_{3v}–Sn_2	7.6044	7.6044	19.0800	955.5170	90	90	120
C_{3v}–Sn_3	7.6040	7.6040	19.0821	955.5352	90	90	120
D_{3d}-Sn_4	7.6240	7.6240	19.0473	958.8152	90	90	120
Experimental	7.3797	7.3797	18.3778	866.76	90	90	120

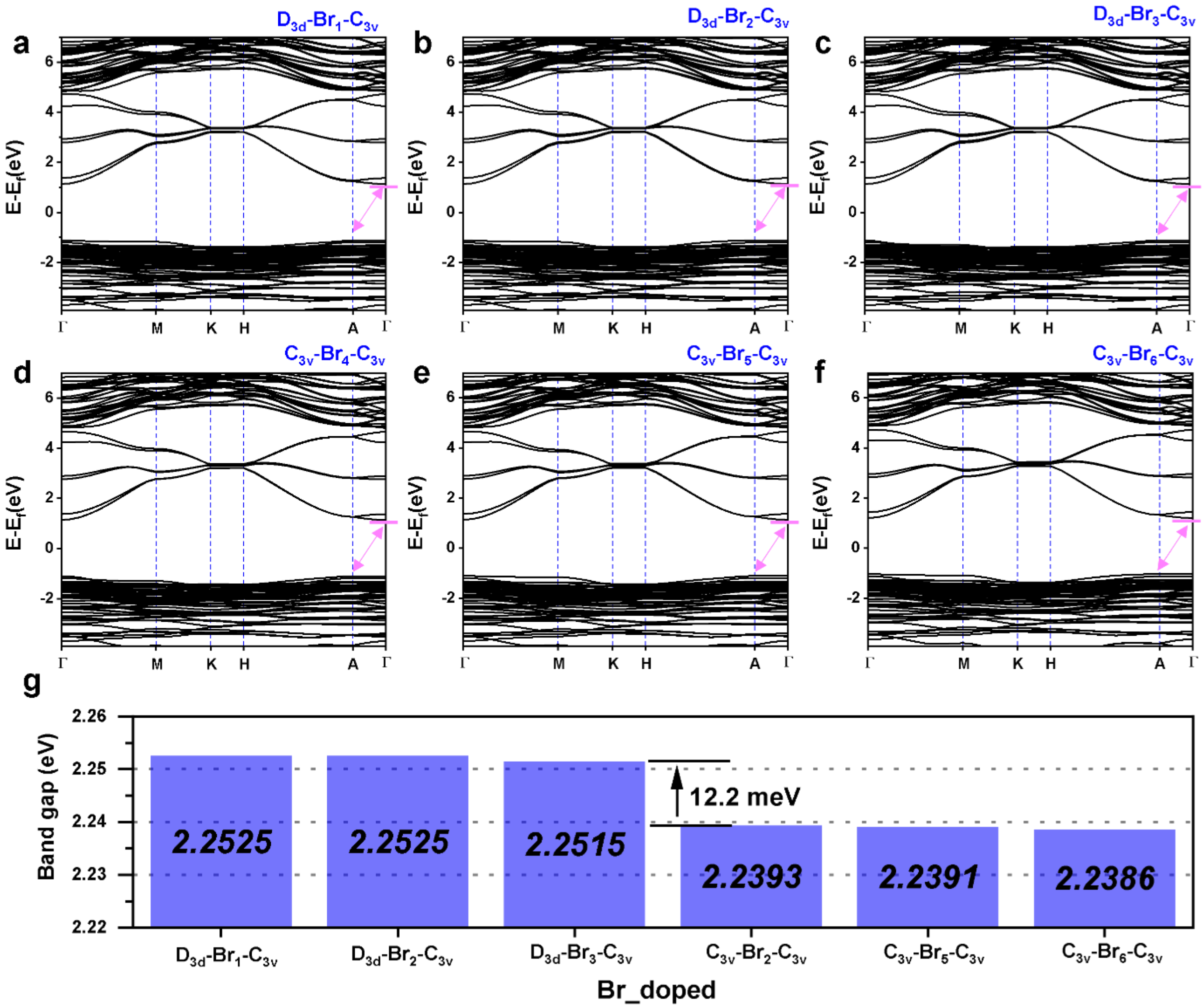

Supplementary Fig. 46 Band structures of the Br-doping model for **a** D_{3d}-Br_1-C_{3v}, **b** D_{3d}-Br_2-C_{3v}, **c** D_{3d}-Br_3-C_{3v}, **d** C_{3v} -Br_4-C_{3v}, **e** C_{3v} -Br_5-C_{3v}, **f** C_{3v} -Br_6-C_{3v}. **g** Band gap for these doping models.

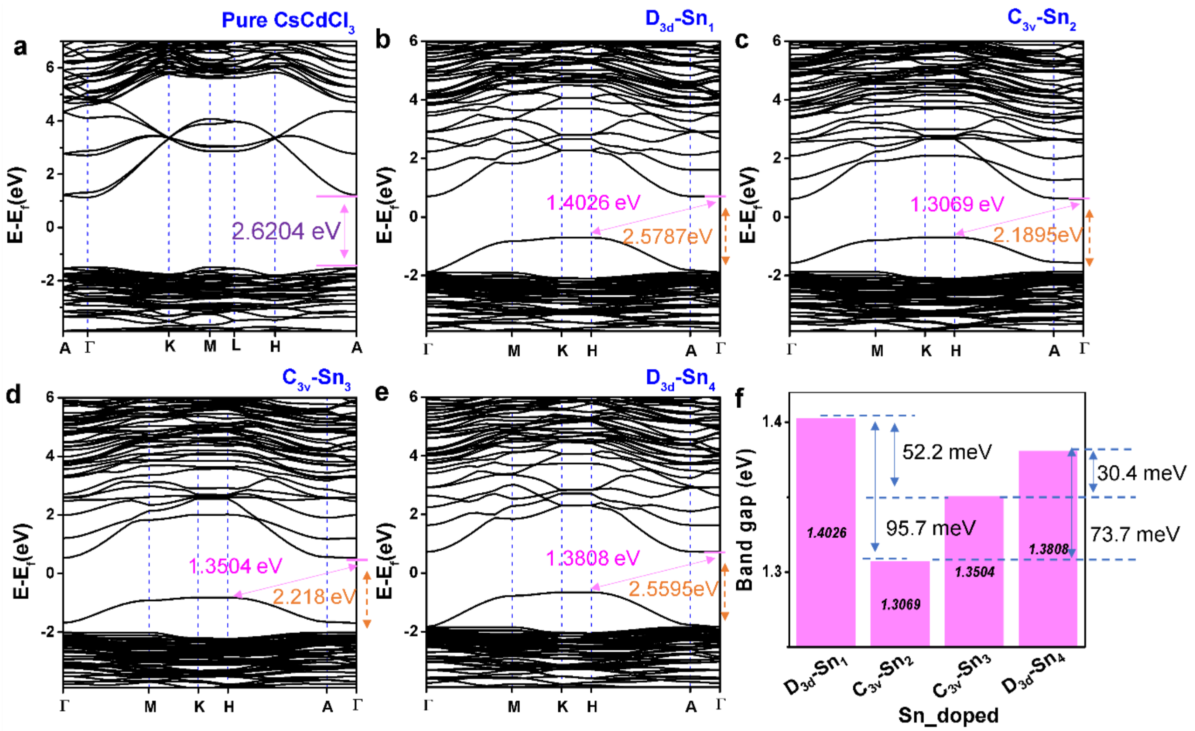

Supplementary Fig. 47 Band structures of **a** pure $CsCdCl_3$ and the Sn-doping model for **b** D_{3d}-Sn_1, **c** C_{3v} –Sn_2, **d** C_{3v}–Sn_3 and **e** D_{3d}-Sn_4. **f** Band gap for these doping models.

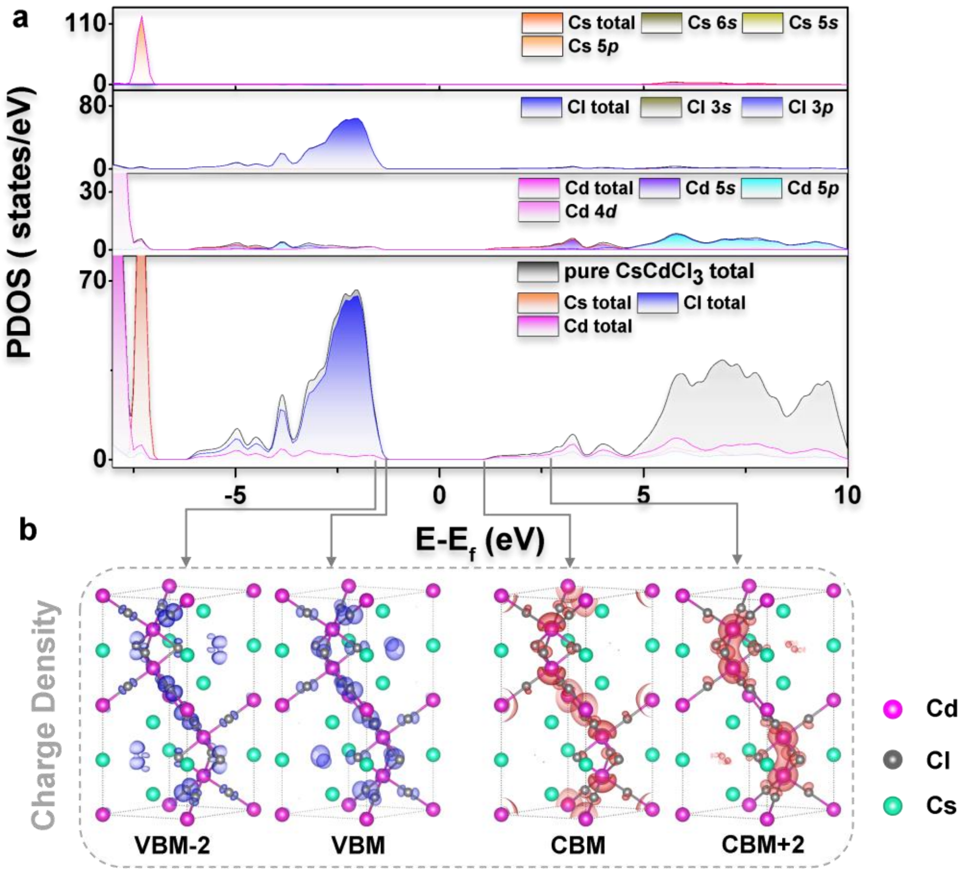

Supplementary Fig. 48 a PDOS and **b** the Gamma point is visualized with VBM and CBM-associated charge density maps of pure $CsCdCl_3$. PDOS: projected density of states.

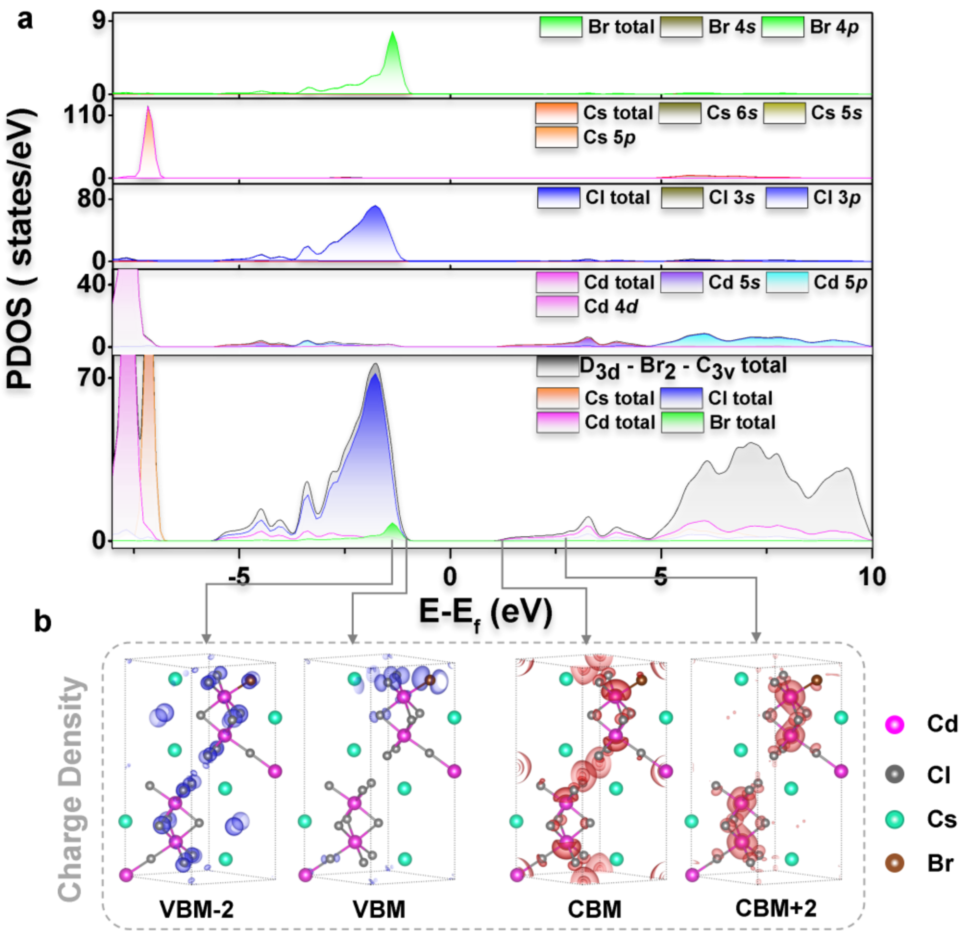

Supplementary Fig. 49 a PDOS and **b** the Gamma point is visualized with VBM and CBM-associated charge density maps of D_{3d}-Br_2-C_{3v} model. PDOS: projected density of states.

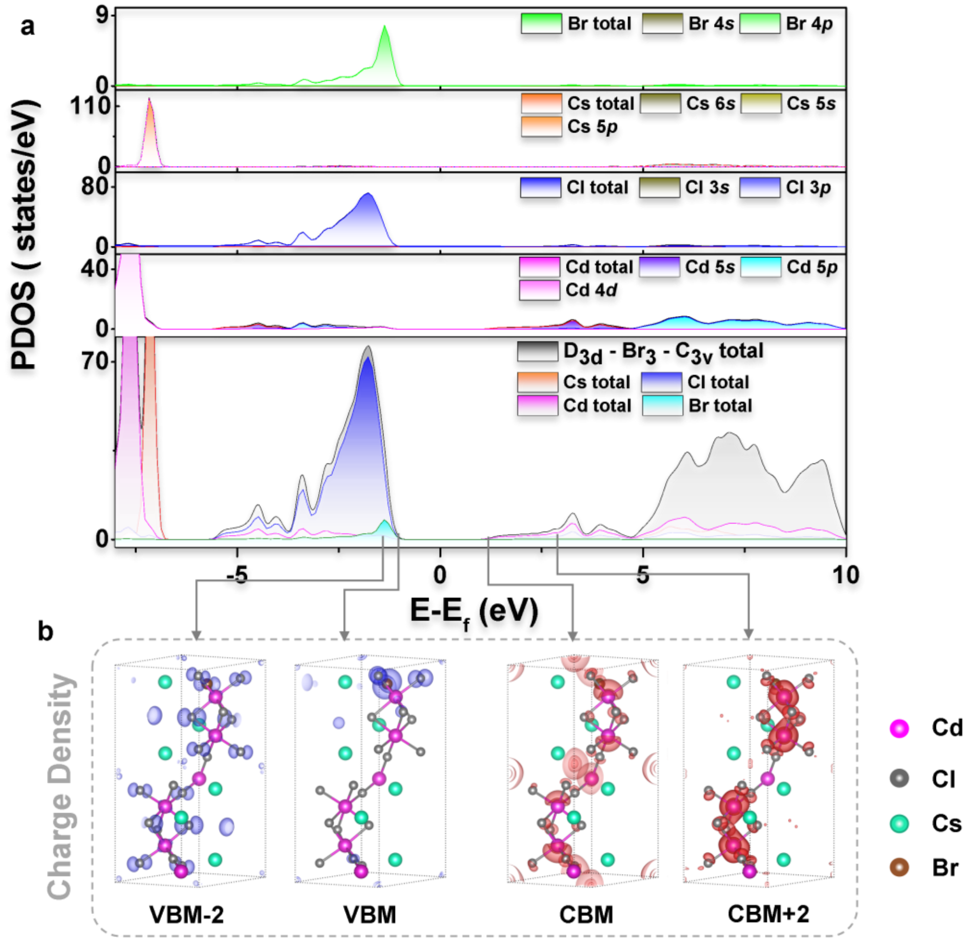

Supplementary Fig. 50 a PDOS and **b** the Gamma point is visualized with VBM and CBM-associated charge density maps of D_{3d}-Br_3-C_{3v} model. PDOS: projected density of states.

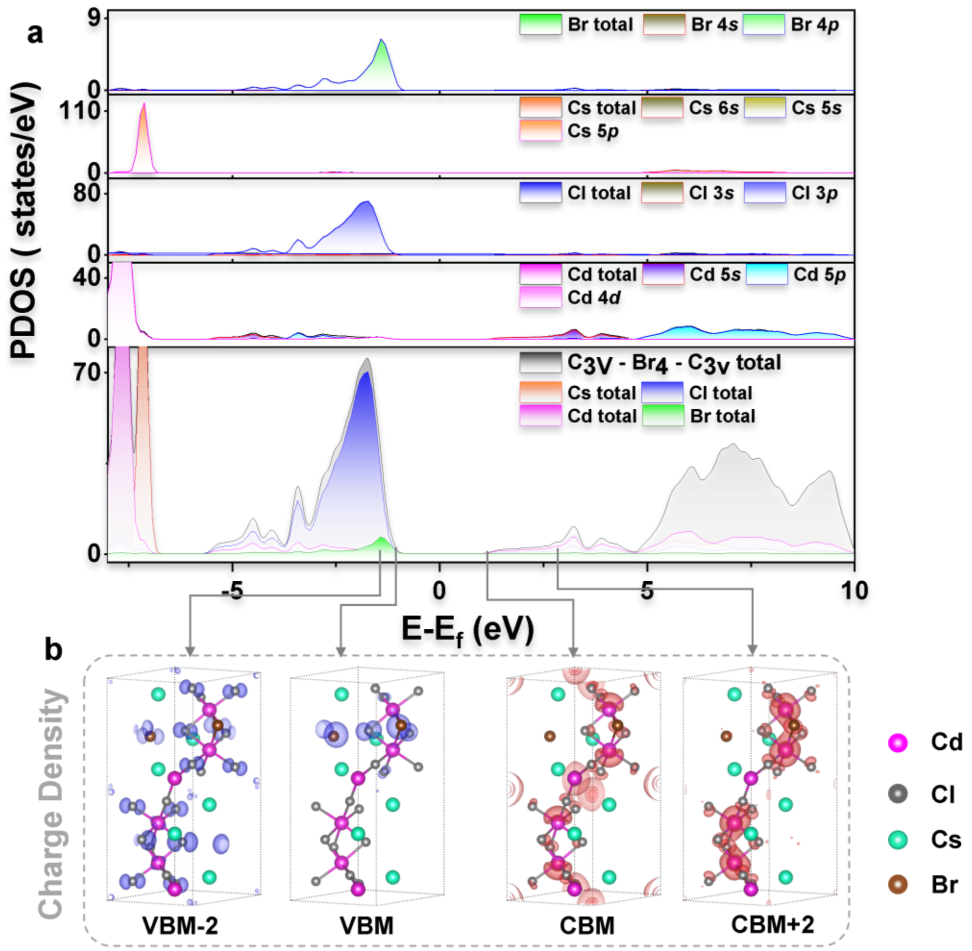

Supplementary Fig. 51 a PDOS and **b** the Gamma point is visualized with VBM and CBM-associated charge density maps of C_{3v}-Br_4-C_{3v} model. PDOS: projected density of states.

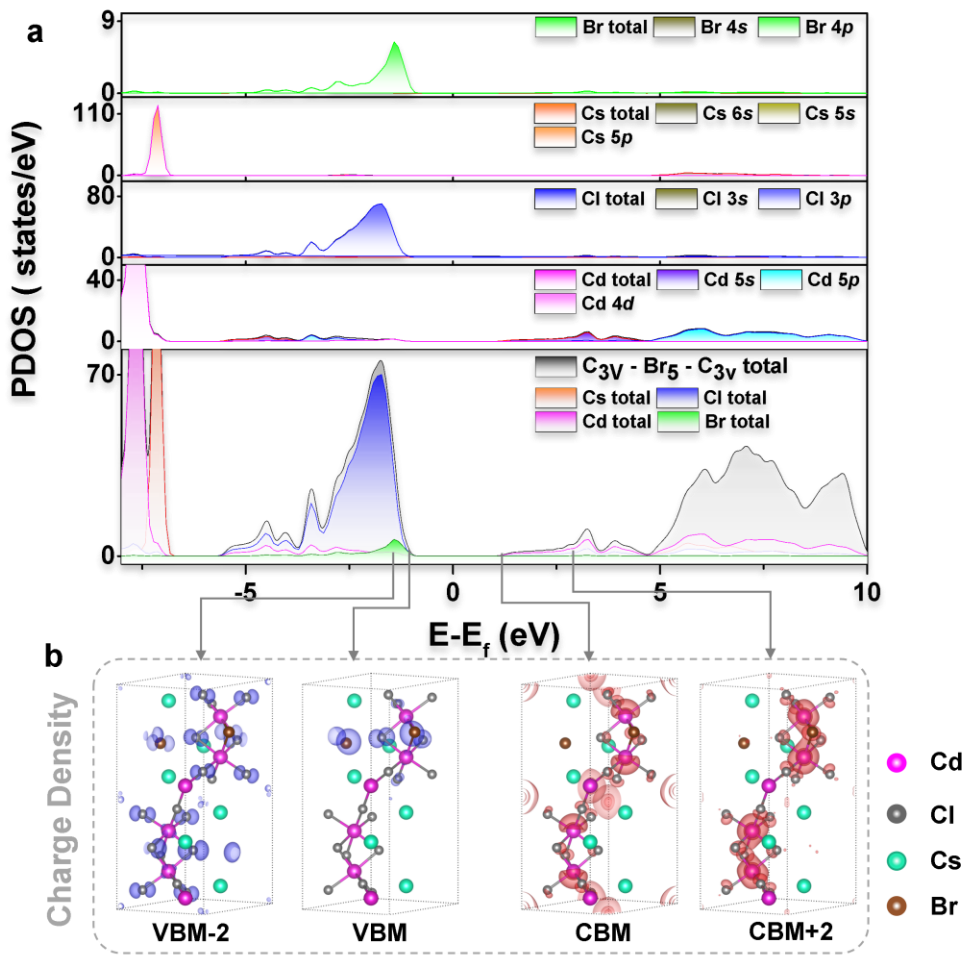

Supplementary Fig. 52 **a** PDOS and **b** the Gamma point is visualized with VBM and CBM-associated charge density maps of C_{3v}-Br_5-C_{3v} model. PDOS: projected density of states.

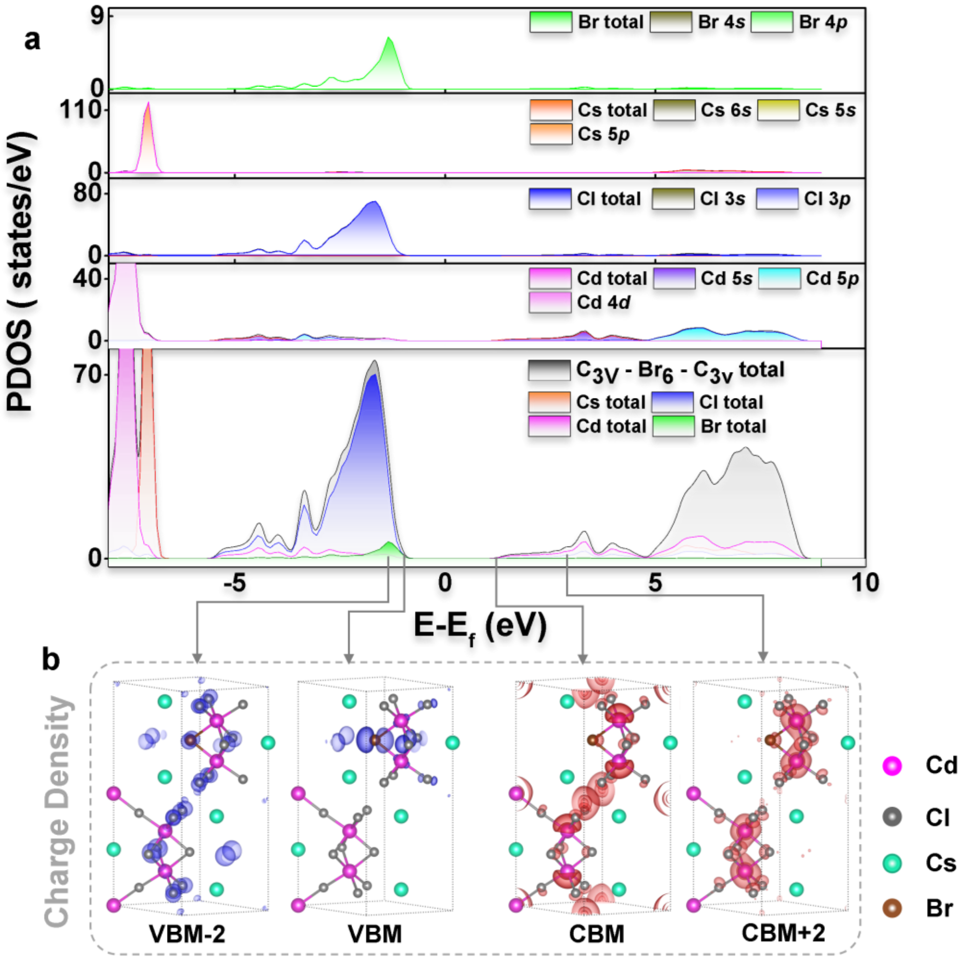

Supplementary Fig. 53 a PDOS and **b** the Gamma point is visualized with VBM and CBM-associated charge density maps of C_{3v}-Br_6-C_{3v} model. PDOS: projected density of states.

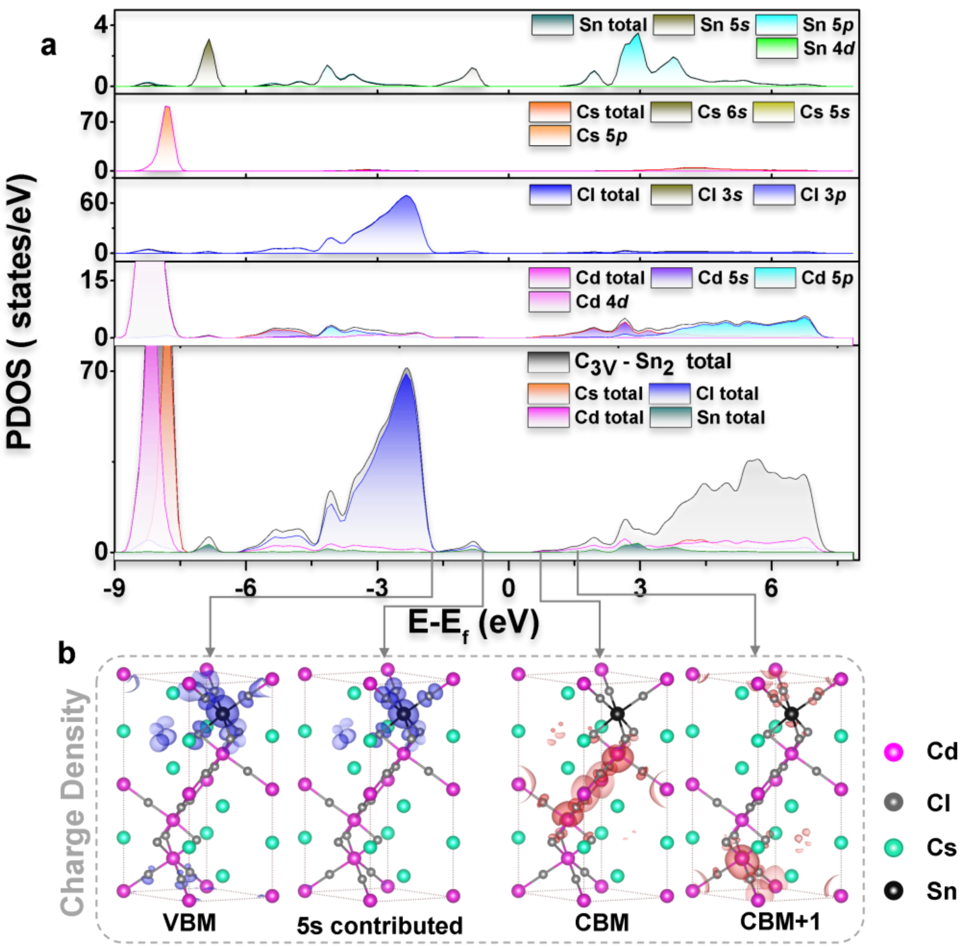

Supplementary Fig. 54 a PDOS and **b** the Gamma point is visualized with VBM and CBM-associated charge density maps in C_{3v}-Sn_2 model, as well as H point for 5*s*(Sn^{2+}) contributed charge density maps. PDOS: projected density of states.

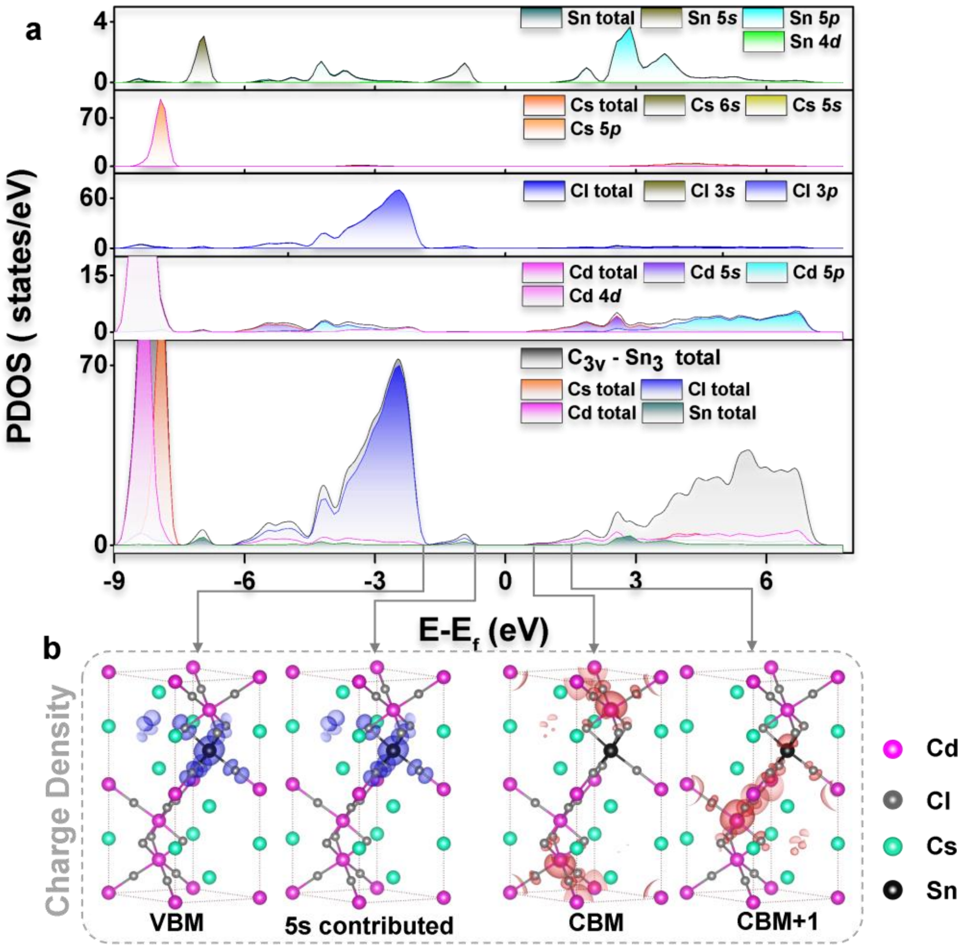

Supplementary Fig. 55 a PDOS and **b** the Gamma point is visualized with VBM and CBM-associated charge density maps in C_{3v}-Sn_3 model, as well as H point for 5*s*(Sn^{2+}) contributed charge density maps. PDOS: projected density of states.

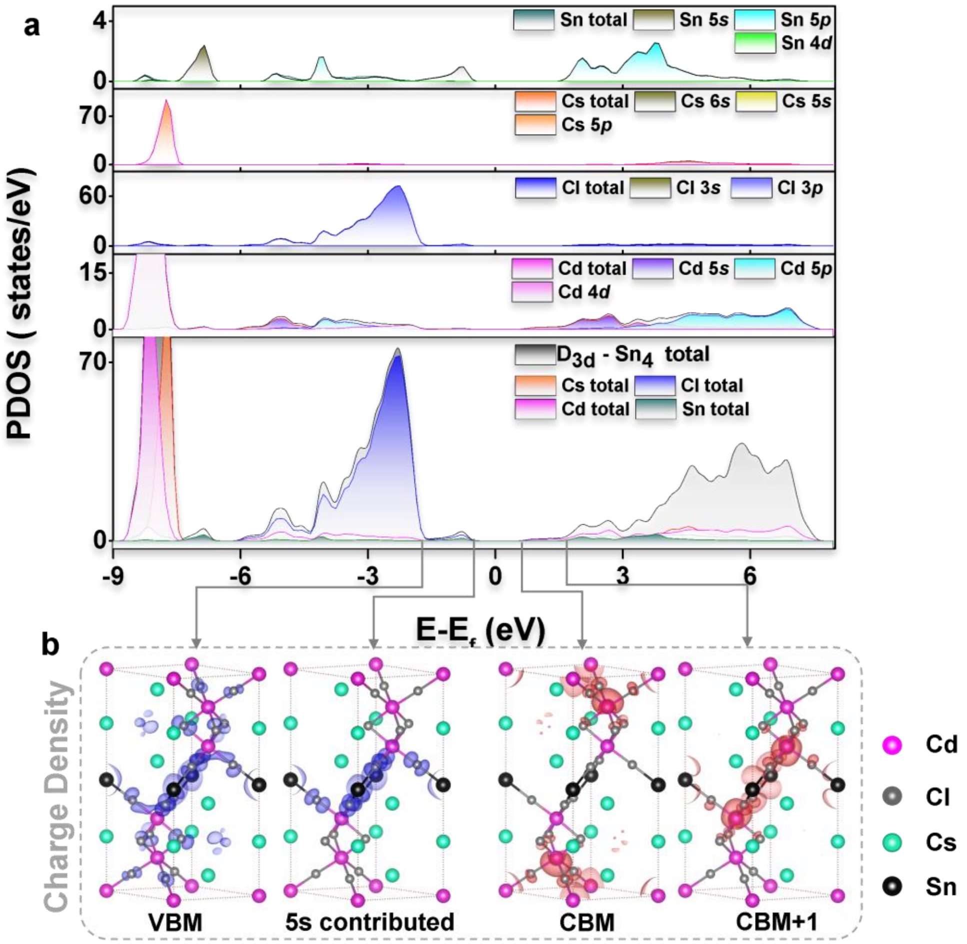

Supplementary Fig. 56 **a** PDOS and **b** the Gamma point is visualized with VBM and CBM-associated charge density maps in D_{3d}-Sn_4 model, as well as H point for 5*s*(Sn^{2+}) contributed charge density maps. PDOS: projected density of states.

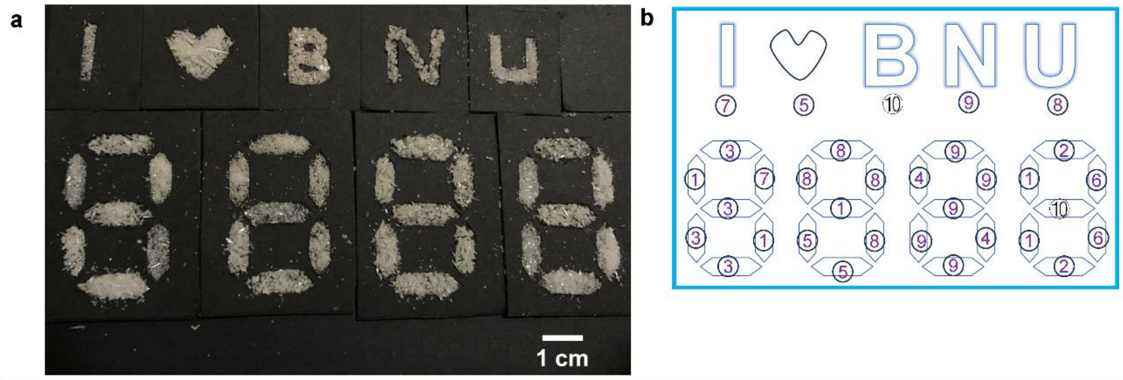

Supplementary Fig. 57. a Photographs of $CsCdCl_3$:*x*%Br and $CsCdCl_3$:*x*%Sn sample powders. **b** Corresponding materials, $CsCdCl_3$:*x*%Br, ①(0%Br), ②(0.2%Br), ③(0.5%Br), ④(0.8%Br), ⑤(1%Br) and ⑥(15%Br); $CsCdCl_3$:*x*%Sn, ⑦(1%Sn), ⑧(3% Sn), ⑨(5% Sn) and ⑩(10% Sn).

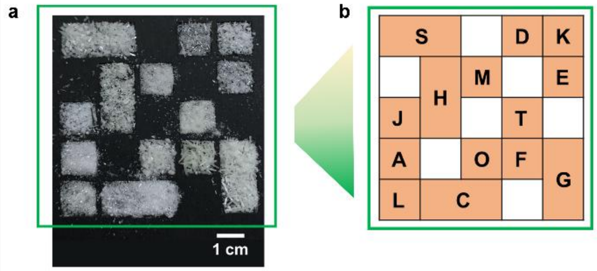

Supplementary Fig. 58. a Photographs of the QR code map includes $CsCdCl_3$:*x*%Br and $CsCdCl_3$:*x*%Sn, **b** with A (0%Br), C(0.2%Br), D (0.5%Br), E (0.8%Br), F (1%Br), G (3%Br), H (5%Br), J (10%Br), H (15% Br), L (1% Sn), M (3% Sn), O (5% Sn), S (10% Sn) and T (15 % Sn).

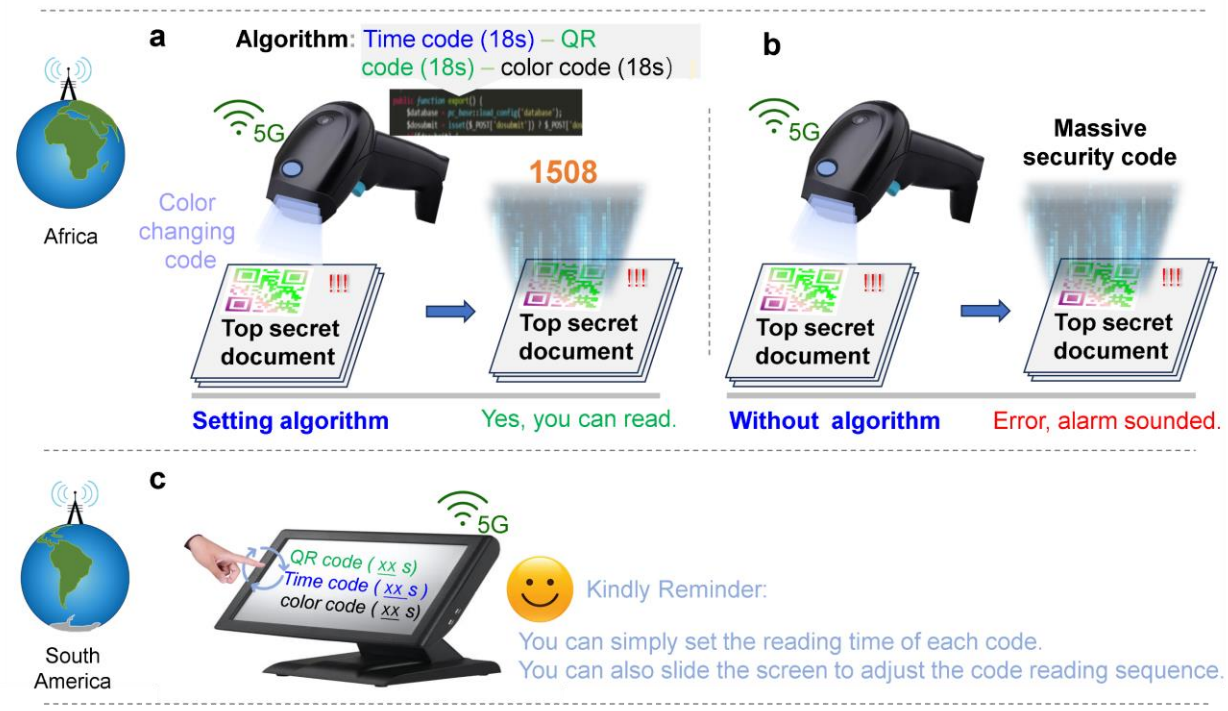

Supplementary Figs 59. a-c Conceptual diagram of 3D code reader and simple algorithm setup process. Note: Leveraging 5G communications, one can establish algorithms across continents to ensure access rights to top-secret documents under their disposal.

Supplementary References

1. Zheng, W. et al. Solution-grown chloride perovskite crystal of red afterglow. *Angew. Chem. Int. Ed.* **60**, 24450 –24455(2021).

2. Liu, N. et al. Near-infrared afterglow and related photochromism from solution-grown perovskite crystal. *Adv. Funct. Mater.* **32**, 2110663(2022).

3. Wang, X. et al. Nearly-unity quantum yield and 12-hour afterglow from a transparent perovskite of $Cs_2NaScCl_6$:Tb. *Angew. Chem. Int. Ed.* **61**, e202210853(2022).

4. He, S. et al. Highly stable orange-red long-persistent luminescent $CsCdCl_3$:Mn^{2+} perovskite crystal. *Angew. Chem. Int. Ed.* **61**, e202208937(2022).

5. Tang, Z. et al. Highly efficient and ultralong afterglow emission with anti-thermal quenching from $CsCdCl_3$: Mn perovskite single crystals. *Angew. Chem. Int. Ed.* **61**, e202210975(2022).

6. Kabe, R. &Adachi, C. Organic long persistent luminescence. *Nature* **550**, 384–387(2017).

7. Lin, Z. et al. Organic long-persistent luminescence from a flexible and transparent doped polymer. *Adv. Mater.* **30**, 1803713(2018).
8. Liang, X. et al. Organic long persistent luminescence through in situ generation of cuprous(I) ion pairs in ionic solids. *Angew. Chem. Int. Ed.* **60**, 24437 –24442(2021).
9. Alam, P. et al. Organic long-persistent luminescence from a single-component aggregate. *J. Am. Chem. Soc.* **144**, 3050−3062(2022).
10. Wu, L. et al. Synthesis and optical properties of a $Y_3(Al/Ga)_5O_{12}:Ce^{3+},Cr^{3+},Nd^{3+}$ persistent luminescence nanophosphor: a promising near-infrared-II nanoprobe for biological applications. *Nanoscale*, **12**, 14180-14187(2020).
11. Zou, R. et al. Magnetic-NIR persistent luminescent dual-modal ZGOCS@MSNs@Gd_2O_3 core–shell nanoprobes for in vivo imaging. *Chem. Mater.* **29**, 3938–3946(2017).
