# Supplementary material for: Full-color, time-valve controllable and Janus-type long-persistent luminescence from all-inorganic halide perovskites
Source: Nat Commun. 2024 Jun 20;15:5281. doi: 10.1038/s41467-024-49654-7 (PMC11190143; doi:10.1038/s41467-024-49654-7)
Supplement: Supplementary file 3 — Description of Additional Supplementary Files [file 41467_2024_49654_MOESM3_ESM.pdf]

Description of Additional Supplementary Files:

Supplementary Movie 1: (.mp4 format). LPL of $CsCdCl3:x\%Br$ and $CsCdCl3:x\%Sn$.

Supplementary Movie 2: (.mp4 format). The progress of 5D anti-counterfeiting.
