# Supplementary material for: Full-color, time-valve controllable and Janus-type long-persistent luminescence from all-inorganic halide perovskites
Source: Nat Commun. 2024 Jun 20;15:5281. doi: 10.1038/s41467-024-49654-7 (PMC11190143; doi:10.1038/s41467-024-49654-7)

checkCIF/PLATON report

Structure factors have been supplied for datablock(s) exp_11624

THIS REPORT IS FOR GUIDANCE ONLY. IF USED AS PART OF A REVIEW PROCEDURE FOR PUBLICATION, IT SHOULD NOT REPLACE THE EXPERTISE OF AN EXPERIENCED CRYSTALLOGRAPHIC REFEREE.

No syntax errors found. CIF dictionary Interpreting this report

Datablock: exp_11624

```
Bond precision:   Cd-Cl = 0.0011 A         Wavelength=0.71073

Cell:               a=7.3797(4)      b=7.3797(4)      c=18.3778(10)
                    alpha=90         beta=90          gamma=120
Temperature:        293 K

                  Calculated                   Reported
Volume            866.77(11)                   866.76(11)
Space group       P 63/m m c                   P 63/m m c
Hall group        -P 6c 2c                     -P 6c 2c
Moiety formula    Cd3 Cl9, 3(Cs)               ?
Sum formula       Cd3 Cl9 Cs3                  Cd0.83 Cl2.48 Cs0.83
Mr                1055.01                      291.03
Dx,g cm-3         4.042                        4.042
Z                 2                            7.250
Mu (mm-1)         11.210                       11.210
F000              924.0                        924.0
F000'             919.83
h,k,lmax          8,8,21                       8,8,21
Nref              334                          329
Tmin,Tmax         0.122,0.106
Tmin'             0.092

Correction method= Not given

Data completeness= 0.985           Theta(max)= 24.969

R(reflections)= 0.0182( 284)                          wR2(reflections)=
                                                      0.0439( 329)
S = 1.214                  Npar= 21
```

The following ALERTS were generated. Each ALERT has the format
test-name_ALERT_alert-type_alert-level.
Click on the hyperlinks for more details of the test.

Alert level C

```
PLAT052_ALERT_1_C Info on Absorption Correction Method   Not Given     Please Do !
PLAT906_ALERT_3_C Large K Value in the Analysis of Variance ......     15.262 Check
PLAT911_ALERT_3_C Missing FCF Refl Between Thmin & STh/L=    0.594          3 Report
```

Alert level G

```
CELLZ01_ALERT_1_G Difference between formula and atom_site contents detected.
CELLZ01_ALERT_1_G ALERT: check formula stoichiometry or atom site occupancies.
           From the CIF: _cell_formula_units_Z    7
           From the CIF: _chemical_formula_sum  Cd0.83 Cl2.48 Cs0.83
           TEST: Compare cell contents of formula and atom_site data

           atom    Z*formula  cif sites diff
           Cd         6.02      6.00    0.02
           Cl        17.98     18.00   -0.02
           Cs         6.02      6.00    0.02
PLAT004_ALERT_5_G Polymeric Structure Found with Maximum Dimension          2 Info
PLAT045_ALERT_1_G Calculated and Reported Z Differ by a Factor ...      0.276 Check
PLAT100_ALERT_5_G A Non-Integer Z value Reported in the CIF ......      7.250 Check
PLAT199_ALERT_1_G Reported _cell_measurement_temperature ..... (K)        293 Check
PLAT200_ALERT_1_G Reported   _diffrn_ambient_temperature ..... (K)        293 Check
PLAT720_ALERT_4_G Number of Unusual/Non-Standard Labels ..........          6 Note
PLAT794_ALERT_5_G Tentative Bond Valency for Cd03      (II)      .       2.12 Info
PLAT794_ALERT_5_G Tentative Bond Valency for Cd04      (II)      .       2.05 Info
PLAT883_ALERT_1_G No Info/Value for _atom_sites_solution_primary .     Please Do !
PLAT899_ALERT_4_G SHELXL2018 is Deprecated and Succeeded by SHELXL     2019/3 Note
PLAT909_ALERT_3_G Percentage of I>2sig(I) Data at Theta(Max) Still        81% Note
PLAT910_ALERT_3_G Missing # of FCF Reflection(s) Below Theta(Min).          2 Note
PLAT913_ALERT_3_G Missing # of Very Strong Reflections in FCF ....          3 Note
PLAT933_ALERT_2_G Number of HKL-OMIT Records in Embedded .res File          3 Note
```

0 **ALERT level A** = Most likely a serious problem - resolve or explain
0 **ALERT level B** = A potentially serious problem, consider carefully
3 **ALERT level C** = Check. Ensure it is not caused by an omission or oversight
16 **ALERT level G** = General information/check it is not something unexpected

7 ALERT type 1 CIF construction/syntax error, inconsistent or missing data
1 ALERT type 2 Indicator that the structure model may be wrong or deficient
5 ALERT type 3 Indicator that the structure quality may be low
2 ALERT type 4 Improvement, methodology, query or suggestion
4 ALERT type 5 Informative message, check

It is advisable to attempt to resolve as many as possible of the alerts in all categories. Often the minor alerts point to easily fixed oversights, errors and omissions in your CIF or refinement strategy, so attention to these fine details can be worthwhile. In order to resolve some of the more serious problems it may be necessary to carry out additional measurements or structure refinements. However, the purpose of your study may justify the reported deviations and the more serious of these should normally be commented upon in the discussion or experimental section of a paper or in the "special_details" fields of the CIF. checkCIF was carefully designed to identify outliers and unusual parameters, but every test has its limitations and alerts that are not important in a particular case may appear. Conversely, the absence of alerts does not guarantee there are no aspects of the results needing attention. It is up to the individual to critically assess their own results and, if necessary, seek expert advice.

Publication of your CIF in IUCr journals

A basic structural check has been run on your CIF. These basic checks will be run on all CIFs submitted for publication in IUCr journals (*Acta Crystallographica*, *Journal of Applied Crystallography*, *Journal of Synchrotron Radiation*); however, if you intend to submit to *Acta Crystallographica Section C* or *E* or *IUCrData*, you should make sure that full publication checks are run on the final version of your CIF prior to submission.

Publication of your CIF in other journals

Please refer to the *Notes for Authors* of the relevant journal for any special instructions relating to CIF submission.

PLATON version of 06/07/2023; check.def file version of 30/06/2023

Datablock exp_11624 - ellipsoid plot

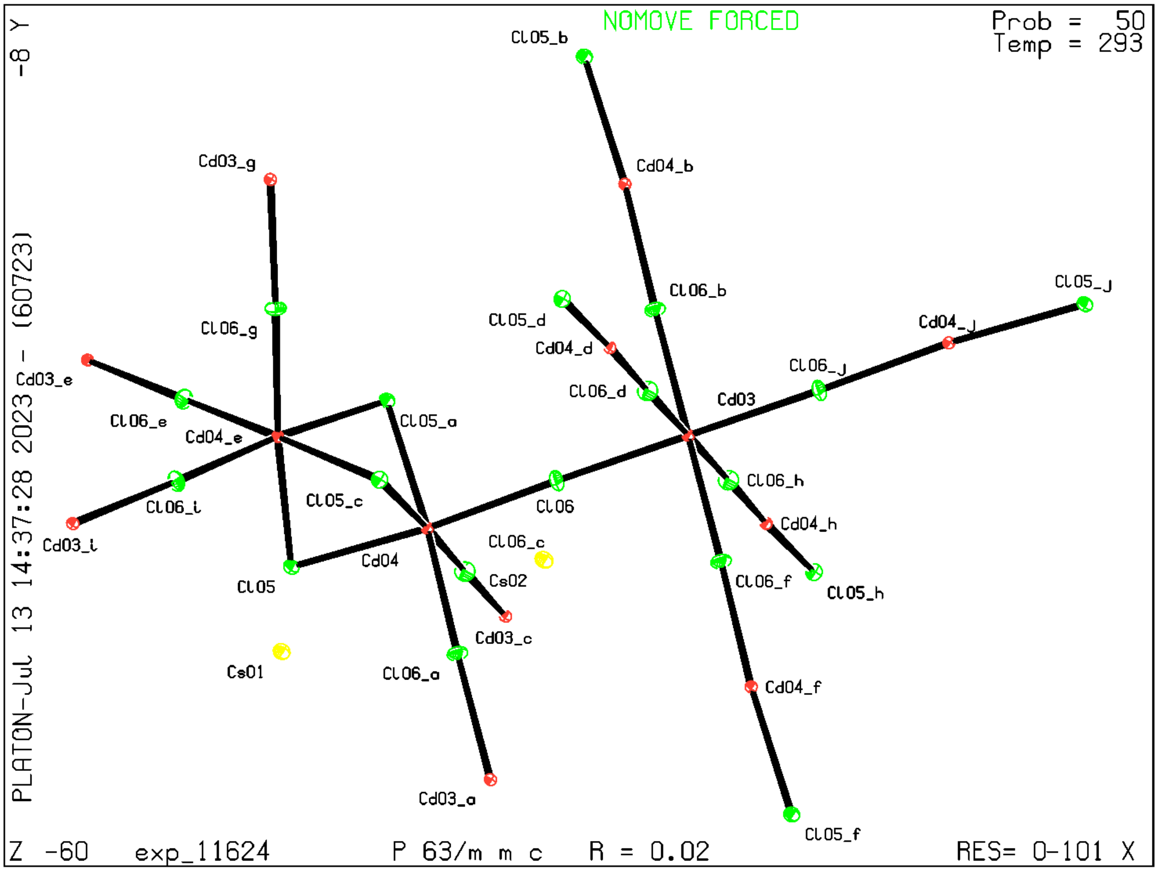

Supplement: Supplementary file 4 — Supplementary Data 1 [file 41467_2024_49654_MOESM4_ESM.pdf]
